# Characterization of the Attachment of Three New Coliphages onto the Ferrichrome Transporter FhuA

**DOI:** 10.1128/jvi.00667-23

**Published:** 2023-06-13

**Authors:** Jessica M. Lewis, Kathryn E. Janda, David B. Kotter, Julianne H. Grose, William R. McCleary

**Affiliations:** a Department of Microbiology and Molecular Biology, Brigham Young University, Provo, Utah, USA; University of Kentucky College of Medicine

**Keywords:** FhuA, phage attachment, coliphage, phage evolution

## Abstract

Receptor-binding proteins (RBPs) allow phages to dock onto their host and initiate infection through the recognition of proteinaceous or saccharidic receptors located on the cell surface. FhuA is the ferrichrome hydroxamate transporter in Escherichia coli and serves as a receptor for the well-characterized phages T1, T5, and phi80. To further characterize how other FhuA-dependent phages attach to FhuA, we isolated and published the genomes of three new FhuA-dependent coliphages: JLBYU37, JLBYU41, and JLBYU60. We identified the egions of FhuA involved in phage attachment by testing the effect of mutant *fhuA* alleles containing single-loop deletions of extracellular loops (L3, L4, L5, L8, L10, and L11) on phage infectivity. Deletion of loop 8 resulted in complete resistance to SO1-like phages JLBYU37 and JLBYU60 and the previously isolated vB_EcoD_Teewinot phage, but no single-loop deletions significantly altered the infection of T1-like JLBYU41. Additionally, lipopolysaccharide (LPS) truncation coupled with the L5 mutant significantly impaired the infectivity of JLBYU37 and JLBYU60. Moreover, significant reductions in the infectivity of JLBYU41 were observed upon LPS truncation in the L8 mutant strain. Analysis of the evolutionary relationships among FhuA-dependent phage RBPs highlights the conservation of L8 dependence in JLBYU37, JLBYU60, Teewinot, T5, and phi80, but also showcases how positive selective pressure and/or homologous recombination also selected for L4 dependence in T1 and even the lack of complete loop dependence in JLBYU41.

**IMPORTANCE** Phage attachment is the first step of phage infection and plays a role in governing host specificity. Characterizing the interactions taking place between phage tail fibers and bacterial receptors that better equip bacteria to survive within the human body may provide insights to aid the development of phage therapeutics.

## INTRODUCTION

The possibility of phage therapy was explored in the early 1900s to treat bacterial infections, but research in the United States ceased with the discovery of antibiotics (1, 2). However, the rise in antibiotic-resistant infections is becoming a major issue, causing a resurgence in phage therapy. In 2019 alone, 1.27 and 4.95 million deaths were attributed to or associated with antibiotic-resistant infections, respectively, and infections caused by Escherichia coli alone were responsible for the most deaths in both cases (3). The number of antibiotic-resistant infections is growing at an alarming rate and mandates the discovery of alternative treatments. Utilization of phage therapeutics is a potential candidate and multiple cases of successful phage therapies have been reported (4–7). However, further understanding of the processes behind a successful phage infection could aid in the development of phage therapy.

Unlike antibiotics, phages can be very selective regarding which hosts they infect. This selection partially occurs at the initial stages of infection during host attachment; however, host defenses such as restriction modification systems (8), CRISPR-Cas (9), and bacterial suicide (10) can also prevent a phage from completing its replication cycle (11). Phage attachment occurs in two binding steps, classified as reversible and irreversible binding (12–15). Specialized receptor-binding proteins (RBPs) located at the tips of phage tail fiber and spike proteins allow them to bind to receptors located on the surface of the bacterial cell. Reversible binding initially occurs on cell structures that are easily accessible, which helps to loosely tether the phage to the cell, but the phage can still detach. Irreversible binding triggers ejection of the genetic material into the cell, and phage replication follows (14, 15). Phages can bind to a single receptor or may require a multiple-receptor mechanism in which the RBPs and receptors used during reversible and irreversible binding are not the same (13). For example, lipopolysaccharide (LPS) has been shown to be highly involved in phage attachment and can be bound either individually or coupled with other receptors (16, 17). Reversible binding to the O-antigen, outer and inner core, or lipid A of LPS can also aid in phage stability and infection efficiency by leading it to the cell surface where irreversible attachment occurs (14, 15). Mutations that eliminate portions of LPS, such as the O-antigen in the MG1655 strain of E. coli or removal of the heptose core in a Δ*waaC* mutant, can reduce or even block phage infection (18, 19). The host range of a phage is highly dependent upon the receptors it targets, and analyzing how tail fibers interact with their receptors is crucial to unlocking the mysteries behind phage host specificity.

Studies have shown that some phages attach to multiple receptors (20–22) or target receptors that could give their host an environmental advantage. For example, iron uptake in Gram-negative cells is mediated through outer membrane porins (23, 24), which play a key role in determining the virulence of human pathogens (25, 26). Phages T1, T5, and phi80 have been shown to adhere to the ferric hydroxamate transporter FhuA (15, 21, 27–30) and may be candidates for treating certain bacterial infections. While FhuA is not unique to pathogenic strains, loss of additional iron transporters does result in a fitness cost when the host is in an iron-limited environment (31).

The structure of FhuA consists of a 22-stranded antiparallel β-barrel (β1 to β22) that is blocked by an N-terminal cork domain. It is also composed of 11 loops (L1 to L11) and 10 turns (T1 to T10) which form at the extracellular and periplasmic faces, respectively. Active transport of ferrichrome (Fc) through FhuA has been shown to bind to L3, L11, β7, and β9 (32, 33) and is dependent on the inner membrane TonB-complex (21). FhuA has also been shown to transport the antibiotics albomycin (34) and rifamycin CGP 4832 (35, 36) and the bacteriocins colicin M and microcin J25 (37). Attachment of phages T1 and phi80 is both FhuA- and TonB-dependent (15), but T5 infection occurs independently of TonB (27). Additional dependence upon TonB could be used to ensure DNA ejection is triggered within live cells, and the conformational changes it confers in FhuA may provide the signal for irreversible phage attachment.

Once the receptor has been bound, the phage ejects its genetic material into the host cell. How this process occurs, however, is completely dependent on the structure of the phage. Tailed phages fall within the Caudoviricetes class and consist of icosahedral heads and tails that can vary in length and complexity. The well-known phages lambda (38), T1 (15), and T5 (27, 28) fall within this class but all contain noncontractile tails. T4 is also a tailed phage, but it has a more complex tail structure that contracts upon host recognition (39, 40). Both contractile and noncontractile phages eject their genetic material into the host cell following irreversible attachment to their receptor(s). Previously, contractile and noncontractile tailed phages were classified as *Myoviridae* and *Siphoviridae*, respectively, but recent updates to phage phylogeny have allowed for phages to be further distinguished (41) and these terms are now used only to refer to the overall structure of the phage. For example, T1-like phages contain noncontractile tails, so it can be said that they have *Siphoviridae* structure. However, further classification categorizes them within the family *Drexlerviridae*, which is further divided into different subfamilies (e.g., *Tunavirinae* and *Tempervirinae*) and genera (*Tunavirus*, *Hanrivervirus*, and *Warwickvirus*) (42).

While some phages have been shown to utilize FhuA as their receptor, relatively few FhuA-dependent phages have been characterized to identify (i) which regions of FhuA are involved in phage attachment or DNA ejection, (ii) the role of LPS in FhuA-dependent phage infection, and (iii) the evolutionary relationships of the RBPs of FhuA-dependent phages. To address this, we constructed six *fhuA* loop deletion strains that contained alterations to L3, L4, L5, L8, L10, and L11. The effect of each mutant allele on phage infection was then explored with and without the presence of the LPS heptose core. We also compared the evolutionary relationships of the RBPs of FhuA-dependent phages and their close relatives to analyze whether they coincided with FhuA loop-dependency results.

This work shows which extracellular loops of FhuA play a role in FhuA-dependent phage attachment as well as whether LPS aids in FhuA discovery in three novel coliphages. We also compare these findings against the phylogenetic relationships of their FhuA-dependent RBPs to highlight changes in selective pressure that contributed to the diversity seen in the binding profiles in FhuA-dependent phages. Further understanding of the components of phage receptors that encode host recognition and how their RBP counterparts evolve over time is key to unlocking the secrets that drive host specificity, and could aid in the exploration of phages as potential therapeutics.

## RESULTS

### FhuA-dependent phage isolation and characterization.

Phages were isolated and purified from raw sewage samples taken along the Utah Wasatch front and tested on E. coli strains containing single-gene knockouts of potential phage receptors. Three FhuA-dependent phages were discovered due to their inability, and then restored ability, to infect a MG1655 Δ*fhuA*::*kan* and a Δ*fhuA*::*kan* pJL002 complement strain, respectively. The *fhuA* complement plasmid (pJL002) was generated by cloning *fhuA* from MG1655 and ligating it into pKG116 via the NdeI and KpnI sites using standard techniques. To aid in the analysis of these phages, we extracted and sequenced their genomes using Illumina technology. *De novo* genome assembly was performed, and gene annotations were assigned by comparing each predicted gene with its codon potential and protein BLASTp results. The published phages are named JLBYU37 (accession no. OK272488), JLBYU41 (OK272479), and JLBYU60 (OK272474) and their genomes are publicly available in the NCBI database.

**FIG 1:**
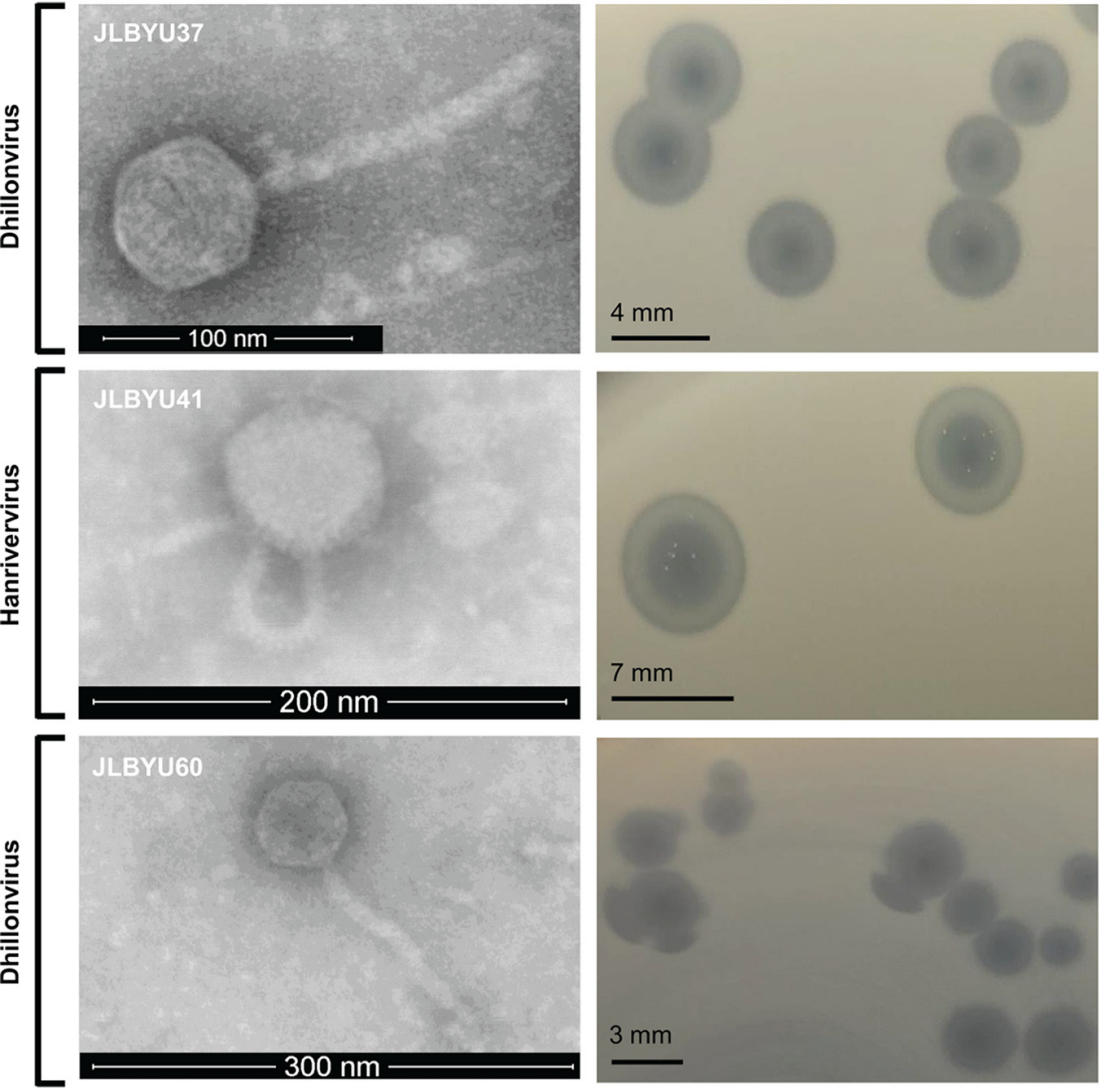
Phage scanning transmission electron microscope images and plaque morphology for JLBYU37, JLBYU41, and JLBYU60. All phages belong to the Caudoviricetes class and have noncontractile tails. JLBYU41 is a T1-like phage that falls within the genus *Hanrivervirus*, but SO1-like phages JLBYU37 and JLBYU60 belong to the genus *Dhillonvirus*. All phages generate bullseye plaques that vary in size, but JLBYU60 generates incomplete halos surrounding the center clearing.

Genome analysis revealed that T1-like phage JLBYU41 belongs to the *Hanrivervirus* genus, whereas JLBYU37 and JLBYU60 fall into the *Dhillonvirus* genus as SO1-like phages (42). This was also supported by scanning transmission electron microscope images of the phage which displayed their *Siphoviridae* structure (Fig. 1). JLBYU37 and JLBYU41 formed 4- and 7-mm bullseye plaques, respectively, but JLBYU60 had plaques that averaged ~3 mm in diameter in LB top agar containing 0.7% agar and consistently contained incomplete halos. All phage plaques were hazy in appearance, suggesting the possibility of a lysogenic lifestyle, but lysogen generation could not be confirmed by standard techniques.

### Genomic and structural protein comparisons.

**FIG 2:**
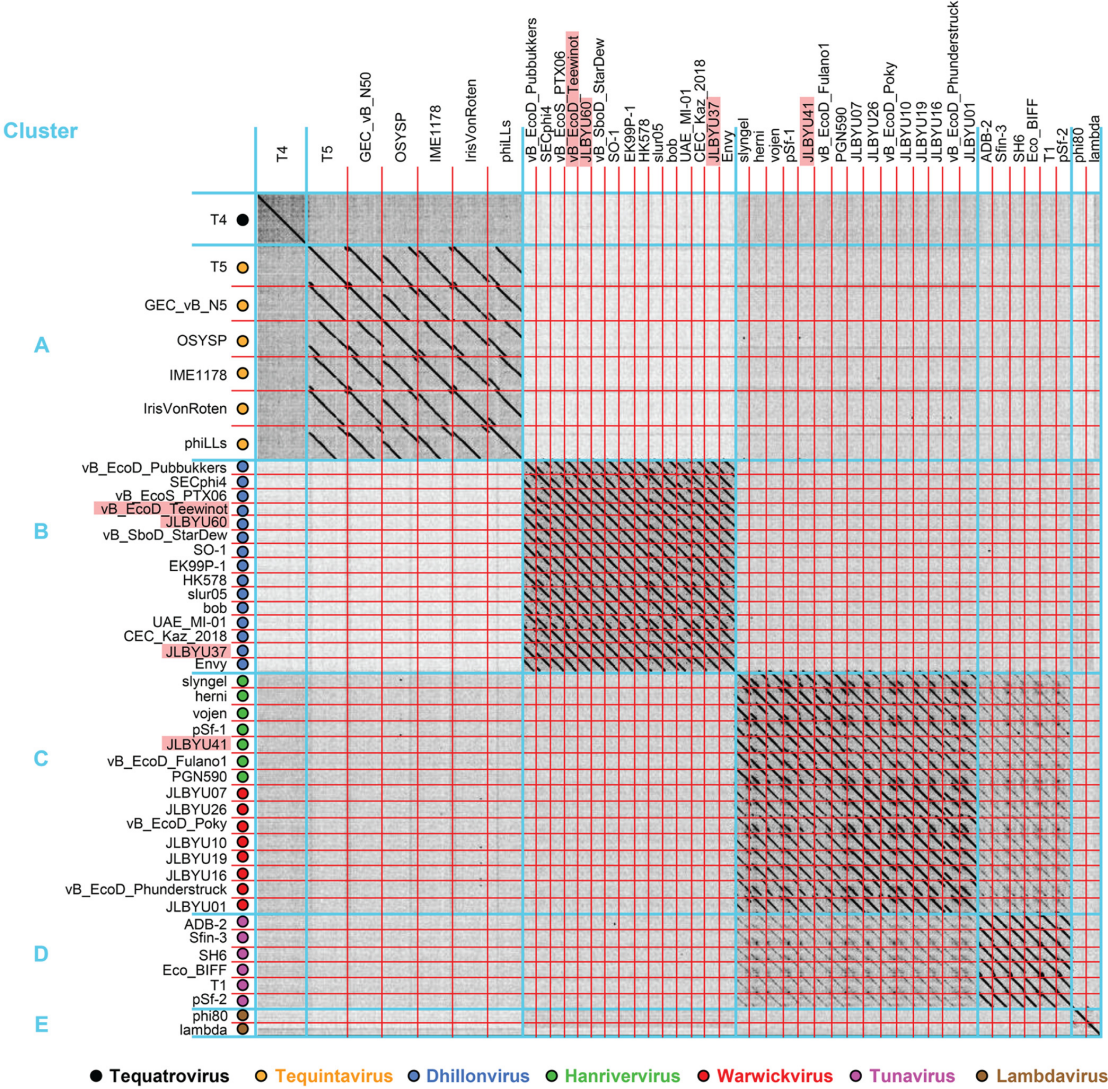
Genome dot-plot analysis of T5-like, T1-like, and SO1-like FhuA-dependent phages. Separation into *Tequatrovirus* (black circle), *Tequintavirus* (orange circles), *Dhillonvirus* (blue circles) and T1-like *Hanrivervirus* (green circles), *Warwickrirus* (red circles), *Tunavirus* (purple circles), and *Lambdavirus* (brown circles) was observed. The names of FhuA-dependent phages identified in this study are highlighted in red, and sequences listed on the vertical axis are compared against themselves on the horizontal axis in the same order. Regions of gene similarity are represented by dots, and diagonal lines suggest high sequence conservation between the compared sequences. Genomes running on the reverse strand were rearranged to provide more accurate alignments, and 5 unique clusters were observed. Tunaviruses resemble hanriverviruses and warwickviruses (cluster C) but were found to be different enough to separate them into their own cluster (D).

**FIG 3:**
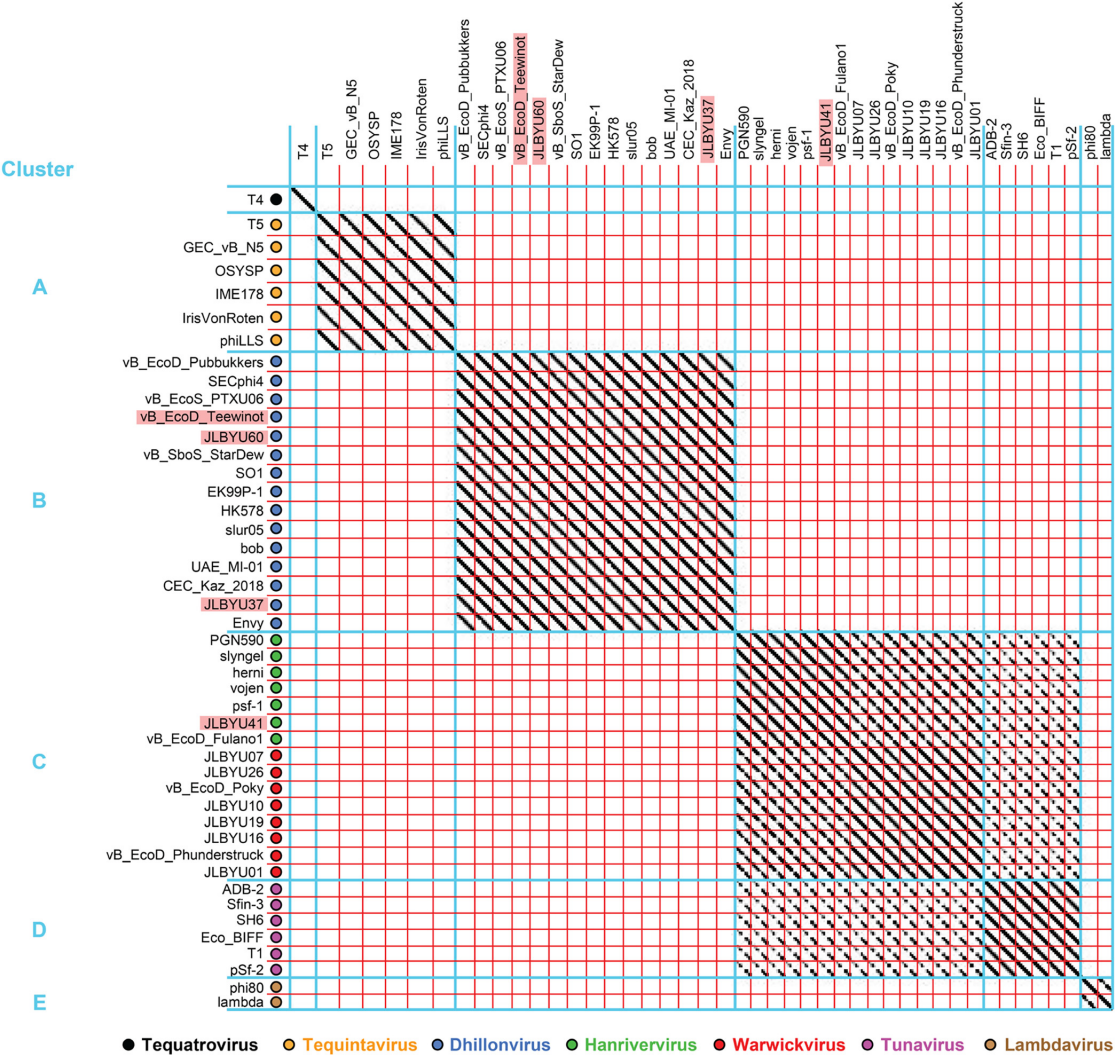
MCP (amino acid) dot-plot analysis of T5-like, T1-like, and SO1-like FhuA-dependent phages. Dot plot appearance is the same as described in the Fig. 2 legend. Comparison of phage MCPs formed the same clusters as shown in the genomic dot-plot analysis.

**FIG 4:**
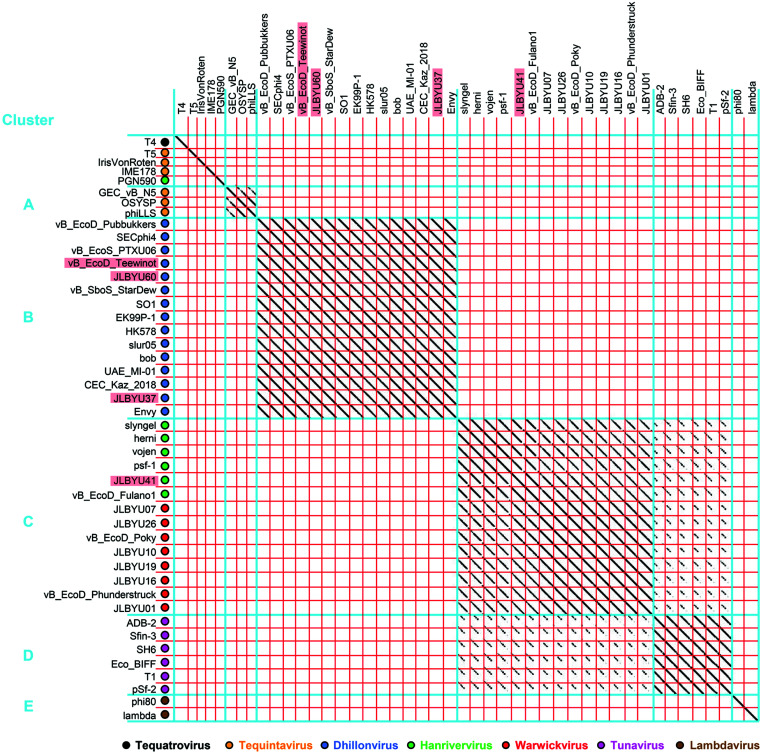
RBP (amino acid) dot-plot analysis of T5-like, T1-like, and SO1-like FhuA-dependent phages. Layout and overall clusters formed in the RBP dot plot mirror those described in the Fig. 2 and 3 legends.

Phage genomes are very mosaic, but structural homology of phage proteins can sometimes be found even when little gene identity is observed. Because of this, relationships within a group of phages may be uncovered using structural data even if there is jumbled identity at the nucleotide level (43). To analyze the conservation of both gene and structural homology among our FhuA-dependent phages, dot-plot comparisons were performed using genomic (nucleotide) sequences (Fig. 2) as well as the protein (amino acid) sequences for the phage major capsid proteins (MCPs) (Fig. 3) and RBPs (Fig. 4). MCPs are more conserved than RBPs and are considered the gold standard when comparing phage relationships. The phages included in this analysis are listed in Table 1 and were pulled from the top MCP BLASTp hits for phages JLBYU37, JLBYU41, and JLBYU60. Previously identified FhuA-dependent phages T1, T5, and phi80 and their top hits were also included, except for the hits from phi80 because they all stemmed from bacterial genomic sequences that most likely harbored lysogenic phage. Since JLBYU37 and JLBYU60 are SO1-like phages and phi80 is a *Lambdavirus*, SO1 and lambda were also included in our analysis. T4 was used as the outgroup because it also falls within the Caudoviricetes class but contains a contractile tail.

**TABLE 1:** Phages analyzed in this study

Phage			Genome		Protein accession no.		Source or reference(s)
Name	Host	Genus	GenBank accession no.	Length (bp)	MCP	RBP	
T4	Escherichia coli	*Tequatrovirus*	NC_000866	168,903	NP_049787.1	NP_049863.1	39, 40
T5	E. coli	*Tequintavirus*	AY543070.1	121,750	YP_006977.1	YP_006985.1	27, 28
IrisVonRoten	E. coli	*Tequintavirus*	MZ501075.1	112,239	QXV80189.1	QXV80353.1	56
GEC_vB_N5	Salmonella enterica	*Tequintavirus*	MW006479.1	110,015	QPI15194.1	QPI15179.1	83
OSYSP	E. coli	*Tequintavirus*	NC_047835.1	110,901	YP_009791015.1	YP_009791005.1	84
phiLLS	E. coli	*Tequintavirus*	NC_047822.1	107,263	YP_009790187.1	YP_009790197.1	85
IME178	E. coli	*Tequintavirus*	MZ398246.1	108,588	QYC97275.1	QYC97266.1	86
PGN590	E. coli	*Hanrivervirus*	NC_049830.1	49,043	YP_009902221.1	YP_009902204.1	NA
vB_EcoD_Pubbukkers	E. coli	*Dhillonvirus*	OK499988.1	44,476	UGO49978.1	UGO49993.1	44
Bob	E. coli	*Dhillonvirus*	MN850628.1	45,256	QHZ59640.1	QHZ59627.1	87
UAE_MI-01	E. coli	*Dhillonvirus*	MW862804.1	44,281	QVD49016.1	QVD49065.1	88
SECphi4	E. coli	*Dhillonvirus*	MT331608.1	44,569	QJI52555.1	QJI52569.1	89
vB_EcoS_PTXU06	E. coli	*Dhillonvirus*	MK373789.1	45,904	QBQ80457.1	QBQ80471.1	90
CEC_Kaz_2018	E. coli	*Dhillonvirus*	MK728541.1	44,283	QCO71631.1	QCO71615.1	91
JLBYU37	E. coli	*Dhillonvirus*	OK272488.1	45,011	UGO56846.1	UGO56832.1	This study
Envy	E. coli	*Dhillonvirus*	NC_031081.1	45,206	YP_009288159.1	YP_009288145.1	92
vB_EcoD_Teewinot	E. coli	*Dhillonvirus*	OK499993.1	41,800	UGO51128.1	UGO51143.1	44
JLBYU60	E. coli	*Dhillonvirus*	OK272474.1	44,804	UGO55266.1	UGO55280.1	This study
vB_SboD_StarDew	Shigella boydii	*Dhillonvirus*	OL615010.1	44,715	UGO46620.1	UGO46634.1	44
SO1	Sodalis glossinidius	*Dhillonvirus*	NC_013600.1	45,169	YP_003344944.1	YP_003344957.1	NA
EK99P-1	*Enterobacteria*/Escherichia	*Dhillonvirus*	NC_024783.1	44,332	YP_009055288.1	YP_009055302.1	93
HK578	E. coli	*Dhillonvirus*	NC_019724	43,741	YP_007112615.1	YP_007112629.1	NA
slur05	E. coli	*Dhillonvirus*	NC_028901.1	43,900	YP_009208112.1	YP_009208099.1	94
phi80	*Enterobacteria*	*Lambdavirus*	NC_021190.1	46,150	AFV29147.1	AFV29161.1	95, 96
Lambda	*Enterobacteria*/Escherichia	*Lambdavirus*	NC_001416.1	48,502	NP_040587.1	NP_040600.1	38
T1	E. coli	*Tunavirus*	NC_005833.1	48,836	YP_003898.1	YP_003912.1	97
Eco_BIFF	E. coli	*Tunavirus*	NC_047996.1	49,372	YP_009803912.1	YP_009803926.1	NA
pSf-2	Shigella flexneri	*Tunavirus*	NC_026010.1	50,109	YP_009112974.1	YP_009112960.1	98
SH6	*Shigella*	*Tunavirus*	NC_047785.1	50,552	YP_009787726.1	YP_009787740.1	99
Sfin-3	S. flexneri	*Tunavirus*	NC_049831.1	50,309	YP_009902253.1	YP_009902267.1	NA
ADB-2	E. coli	*Tunavirus*	NC_019725.1	50,552	YP_007112707.1	YP_007112723.1	100
Slyngel	S. enterica	*Hanrivervirus*	NC_049821.1	51,048	YP_009901481.1	YP_009901494.1	NA
Herni	E. coli	*Hanrivervirus*	NC_049823.1	50,971	YP_009901692.1	YP_009901705.1	NA
Vojen	E. coli	*Hanrivervirus*	NC_049824.1	50,709	YP_009901748.1	YP_009901761.1	NA
pSf-1	S. flexneri	*Hanrivervirus*	NC_021331.1	51,821	YP_008059790.1	YP_008059804.1	101
JLBYU41	E. coli	*Hanrivervirus*	OK272479.1	51,277	UGO55928.1	UGO55941.1	This study
vB_EcoD_Fulano1	E. coli	*Hanrivervirus*	OL539459.1	51,759	UGV22610.1	UGV22624.1	102
JLBYU07	E. coli	*Warwickvirus*	OK272485.1	50,290	UGL62335.1	UGL62349.1	This study
JLBYU26	E. coli	*Warwickvirus*	OK272483.1	51,253	UGO56477.1	UGO56463.1	This study
vB_EcoD_Poky	E. coli	*Warwickvirus*	OL539445.1	51,374	UGO52247.1	UGO52261.1	102
JLBYU10	E. coli	*Warwickvirus*	OK272486.1	50,987	UGL62423.1	UGL62437.1	This study
JLBYU19	E. coli	*Warwickvirus*	OK272489.1	49,897	UGO56933.1	UGO56947.1	This study
JLBYU16	E. coli	*Warwickvirus*	OK272471.1	49,995	UGO54810.1	UGO54824.1	This study
vB_EcoD_Phunderstruck	E. coli	*Warwickvirus*	OL539446.1	51,274	UGO52328.1	UGO52343.1	102
JLBYU01	E. coli	*Warwickvirus*	OK272478.1	51,210	UGO55858.1	UGO55873.1	This study

Dot-plot comparisons of the genomes of T5-like (here, cluster A) and SO1-like phages (cluster B) each separate into 1 cluster, but T1-like phages divide into clusters C and D (Fig. 2). Phage JLBYU41 falls within the *Hanrivervirus* genus (cluster C) of T1-like phages; however, a visible distinction is observed compared to the *Tunavirus* genus (cluster D). No phages showed nucleotide sequence homology with the well-studied phages phi80, lambda, or T4 except for slight homology between lambdaviruses phi80 and lambda (cluster E).

To further understand structural and functional similarities between these phages, dot-plot comparisons of the amino acid sequences of their predicted MCPs (Fig. 3) and RBPs (Fig. 4) were also generated using the same set of phages. MCP analysis revealed high protein sequence conservation within each genus, but various levels of homology occurred between T1-like Hanriverviruses, Warwickviruses and Tunaviruses. The clusters generated in the MCP comparison mirrored those found in the genomic comparisons. Comparisons of their predicted RBPs separated into 4 clusters but T5, IrisVonRoten, IME178 and PGN590 no longer showed sequence similarity with any other phages. Homology between clusters C and D was still noted but similarities vanished when the C-terminal regions of *Tunavirus* RBPs were compared.

The diversity of our FhuA-dependent phages was further characterized by using BLAST and BLASTp to compare the sequence identity of their genomes, MCPs, and RBPs. The genomes of SO1-like phages JLBYU37 and JLBYU60 are 93.45% identical, with 98.36% and 99.30% identity shared between their MCPs and RBPs, respectively. This analysis led to the detection of another FhuA-dependent SO1-like phage previously isolated from our institution called Teewinot (44). The predicted RBP for Teewinot is 99.21% identical to that of JLBYU37 (gp22) and 98.68% identical to that of JLBYU60 (gp24). Genomic comparisons of JLBYU37 and JLBYU60 with Teewinot revealed sequence identities of 97.06% and 93.81%, respectively.

### Classification of FhuA loop dependency.

FhuA complement strains containing single-loop deletions for loops 4, 5, 8, 10, and 11 were designed to replace the same residues as outlined by Endriss et al. (33) and are summarized in Table S1 in the supplemental material. Each loop was replaced with a short peptide chain to reduce the effect that deletions would have on the structure of the β-barrel. A loop 3 deletion mutant was also made, but modifications to the Endriss design were made to shift the site for deletion to begin with L247 to conserve Fc binding sites Y244 and W246 (33). To confirm the availability and functionality of the FhuA loop deletions in the outer membrane, each mutant was tested with a Fc growth promotion assay.

Complementation of *fhuA* in strain MG1655 Δ*fhuA*::*kan* with pJL002 resulted in growth promotion, but normal growth also occurred in the Δ*fhuA*::*kan* pKG116 (empty vector control) when Fc was added as the only iron source. Reduction and restoration of growth could only be observed in Δ*fhuA*::FRT Δ*fepA*::*kan* pKG116 and Δ*fhuA*::FRT Δ*fepA*::*kan* pJL002, respectively, due to the removal of an additional iron transporter FepA. When tested in the Δ*fhuA*::FRT Δ*fepA*::*kan* background, *fhuA* mutant alleles for L4, L5, L8, and L10 (pJL005 to pJL008) displayed growth enhancement. Although L3 and L11 (pJL004 and pJL009) did not show growth enhancement, these mutant proteins are thought to still be integrated into the outer membrane because they restored sensitivity to FhuA-dependent phages compared to the empty vector control.

**FIG 5:**
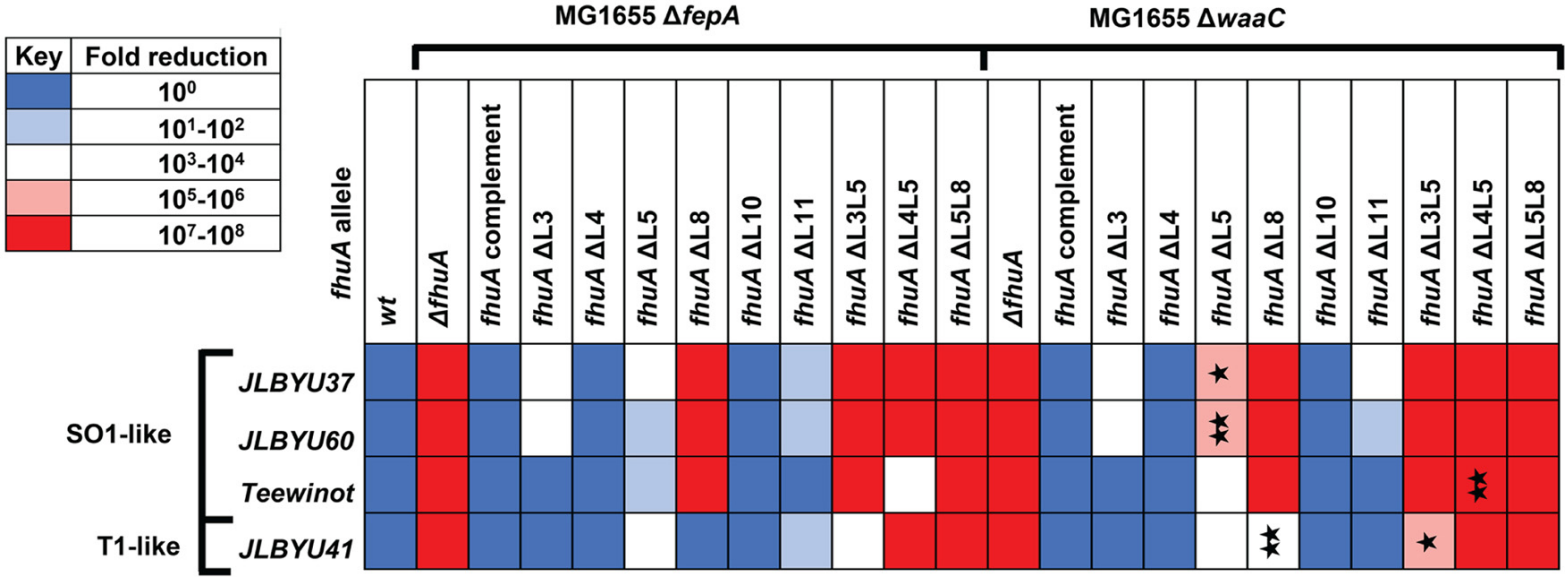
Characterization of FhuA loops utilized during FhuA-dependent phage infection. Effects of single and double FhuA loop deletions were tested against FhuA-dependent phages JLBYU37, JLBYU60, Teewinot, and JLBYU41 in both a MG1655 Δ*fhuA*::FRT Δ*fepA*::*kan* and MG1655 Δ*fhuA*::FRT Δ*waaC*::*kan* background strain. Fold reductions in the LPS mutant strain where *P* < 0.05 or < 0.005 compared to the Δ*fhuA*::FRT Δ*fepA*::*kan* strain are indicated by one and two stars, respectively. Significance was calculated using a Student’s *t* test with two-tailed distribution and two-sample equal variance.

Analysis of the effect of FhuA loop deletions on phage infection revealed the necessity of L8 in the infection of SO1-like phages JLBYU37, JLBYU60, and Teewinot (Fig. 5). Individual L3 and L5 mutants showed a moderate role in the infection of JLBYU37 and JLBYU60, but Teewinot and JLBYU41 were only affected by the L5 mutant. To discover the possible role of LPS in infection efficiency, phage sensitivity was also analyzed in a Δ*fhuA*::FRT Δ*waaC*::*kan* strain expressing each of the *fhuA* mutant alleles (pJL003 to pJL009). As a result, the removal of the inner and outer core of LPS significantly affected the adsorption of phages JLBYU37 and JLBYU60 when L5 was removed (noted by one and two stars in Fig. 5, respectively) but the infection of JLBYU41 was significantly impacted by the L8 mutant (noted by two stars in Fig. 5).

To explore the possibility that JLBYU41 utilizes multiple extracellular loops during attachment, we constructed double-loop deletion mutants L3L5, L4L5, and L5L8 (pJL010 to pJL012) and individually tested for phage infectivity in both the Δ*fhuA*::FRT Δ*fepA*::*kan* and Δ*fhuA*::FRT Δ*waaC*::*kan* backgrounds. Fc growth enhancement was observed for each of the double-mutant strains. As predicted, the L5L8 double-loop deletion mutants were completely resistant to JLBYU37, JLBYU60, and Teewinot, but the mutation also conferred full resistance to JLBYU41. Interestingly, the L4L5 mutant was resistant against all phages except for Teewinot. Removal of the LPS heptose core, however, completely blocked Teewinot infection in the L4L5 mutant. Only JLBYU41 could infect the L3L5 deletion strain, but LPS truncation resulted in significantly decreased infectivity (noted by 1 star in Fig. 5).

### Evolution of FhuA-dependent phages.

Phages are constantly in an evolutionary arms race to maintain their ability to infect their hosts, and one way a phage can evolve is by acquiring changes in its RBPs. Generally, phage RBPs have higher levels of conservation in their N termini but show greater diversity at the C-terminal end (45) suggesting that the C terminus may be under positive selective pressure or undergo homologous recombination (46). Because of this, it can be difficult to understand phage relatedness based upon nucleotide sequences alone. Some phages, however, maintain structural similarities even when there is little homology at the nucleotide level (47–49). Consequently, our phylogenetic analysis was performed using the amino acid sequences for both the RBPs and MCPs in each phage, the latter of which are highly conserved. Although MCPs and RBPs are both structural proteins, we would expect to see altered evolutionary paths due to the role RBPs can play in phage evolution. Comparisons between these trees may reveal unique evolutionary relationships that may appear due to changes in selective pressure or RBP recombination, and may give insight into the role tail fiber evolution may have played in diversifying FhuA-dependent phage attachment. To do this, we performed phylogenetic analysis using the same set of predicted phage MCPs and RBPs that were analyzed in our dot-plot comparisons (See Table 1).

**FIG 6:**
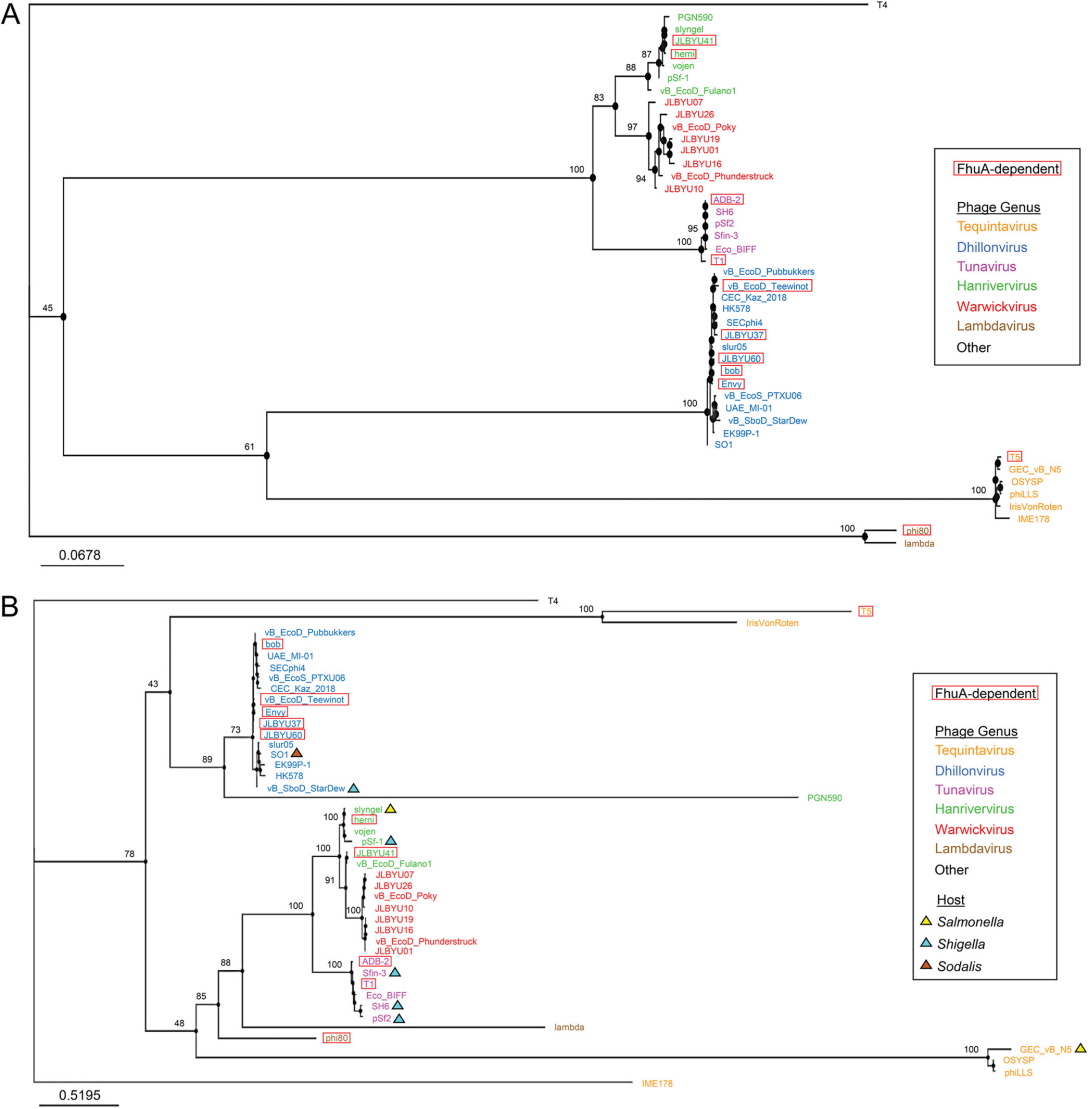
Evolutionary analysis of structural proteins in FhuA-dependent T5, T1-like, and SO1-like phages. Phylogenetic trees for phage MCPs (A) and RBPs (B) were constructed using their protein (amino acid) sequences. Known FhuA-dependent phages are outlined with a red box and *Tequintavirus* (orange), *Dhillonvirus* (blue), *Tunavirus* (purple), *Hanrivervirus* (green), *Warwickvirus* (red), and *Lambdavirus* (brown) are indicated by color. Phages reported to infect Salmonella, *Shigella*, and *Sodalis* in NCBI are indicated by yellow, cyan, and brown triangles, respectively, on the RBP tree. Phages without a triangle were listed or shown to infect E. coli.

The predicted evolutionary relationships for JLBYU37, JLBYU60, Teewinot, and JLBYU41 are represented by a tree generated from their MCP sequences in Fig. 6A. As expected, each phage clusters with members of its respective genus with T1-like Warwickviruses, Hanriverviruses and Tunaviruses diverging early from the ancestors of Tequintaviruses and Dhillonviruses. The ancestor giving rise to Lambdaviruses phi80 and lambda branched from the lineage that gave rise to all T1-like, T5-like and SO1-like phages.

Analysis of the evolution of their putative RBPs, however, suggests a different evolutionary history (Fig. 6B). One of the most noticeable differences is the distribution of *Tequintavirus* phages throughout, which are monophyletic in the MCP tree. *Tequintavirus* IME178 branched off very early from the ancestor that gave rise to *Dhillonvirus*, *Lambdavirus*, T1-like phages, and the remaining tequintaviruses in this analysis. The RBPs of *Dhillonvirus* form a clade with PGN590 and branch off from the ancestor giving rise to T5 + IrisVonRoten tequintaviruses. This varies from the relationships observed for their MCPs, which show *Dhillonvirus* and *Tequintavirus* as sister taxa. GEC-vB_N5, OSYSP, and phiLLS tequintaviruses branch off from the clade giving rise to lambdaviruses and T1-like phages. Lambda and phi80 are more distantly related to each other here than in the MCP tree, which depicts them as sister taxa, but are more closely related to tequintaviruses and T1-like phages. This coincides with their corresponding receptors since lambda binds to the LamB receptor and phi80 is FhuA-dependent, like some other T1-like phages. Warwickviruses and tunaviruses are monophyletic, which is consistent with the findings from the MCP tree, but *Hanrivervirus* RBPs are now paraphyletic. Importantly, although the clade containing FhuA-dependent phage JLBYU41 shares a node with warwickviruses, none of the warwickviruses isolated in our lab (JLBYU01, JLBYU07, JLBYU10, JLBYU16, JLBYU19, or JLBYU26) use FhuA as their sole receptor (personal observation). The differences between the MCP and RBP trees suggest that known evolutionary processes (e.g., recombination or positive selective pressure) could have altered receptor specificity.

To determine the role the C-terminal RBP sequences may have had on these observed differences, we also constructed a phylogenetic tree for the C-terminal region of the RBPs (Fig. S1). The C-terminal start site was chosen by first comparing the RBPs from FhuA-dependent T5-like, T1-like, and SO1-like phages with their homologs in a multiple sequence alignment (not shown). RBP length varied from 585 to 1,192 amino acids and the sequence identities between all RBP sequences were very different, so sequence identity could only be compared between phages within the same genus. The cutoff for the C-terminal start site for each comparison was chosen to be upstream of the region where a reduction in percent identity was observed, and is outlined in Table S3. The C-terminal RBP tree was then constructed as previously described. We then performed a log-likelihood test on the C-terminal RBP tree to determine whether there were any significant changes in the predicted phylogeny compared to the full RBP tree. This was done using IQ-TREE web server topology evaluation with 10,000 replicates. Significant differences were found using the one-sided Kishino-Hasegawa (*P* = 0.0004), Shimodaira-Hasegawa (*P* = 0.0004), and approximately unbiased tests (*P* = 3.03 × 10^−6^). As a control, we also ran this evaluation on the full-length RBP tree, and all tests resulted in no significant differences (*P* = 1). This suggests that natural processes of phage evolution (such as positive selective pressure or homologous recombination) involving the C-terminal regions of the phage RBPs have occurred throughout their evolutionary history and may be responsible for the observed differences in FhuA-dependent phage attachment.

## DISCUSSION

### Effect of FepA on Fc uptake.

In this paper, we analyzed the binding of three novel phages with the FhuA protein from E. coli by characterizing which regions of FhuA are required for a successful phage infection and whether the presence of the LPS heptose core contributes to infection efficiency. At the heart of our analysis was a comparison of the ability of these phages to infect different versions of FhuA with mutated extracellular loops and also to probe the role of LPS in infection. We also analyzed the potential lineage of FhuA-dependent RBPs and its correspondence with FhuA-dependent phage binding profiles.

To do this, we first analyzed the effect of mutant *fhuA* constructs containing single-loop deletions on phage infection. Because loop deletions could potentially disrupt protein stability or membrane insertion, we developed a Fc growth promotion assay to check each mutant for membrane integration, FhuA functionality, and phage accessibility. As a negative control for these experiments, we observed a reduction of growth in a strain harboring an empty vector that did not encode *fhuA*. This loss of growth enhancement was only observed when *fepA* (ferric enterobactin transporter) was also removed. Enterobactin is another siderophore that has been shown to bind ferric iron with high affinity and utilizes the outer membrane transporter FepA. Although FepA is predicted to be ~11× less abundant than FhuA (50), these results suggest that enterobactin may be able to steal enough ferric iron from dipyridyl (an iron chelator that was added to the M9 plates to bind any remaining free iron) or Fc to support normal growth in the Δ*fhuA*::*kan* strain. Possible explanations for this observed bypass of FhuA dependence are (i) the presence of another siderophore which has a higher affinity for iron and can transport stolen iron from Fc or dipyridyl, or that (ii) enough iron remained in the plates to support regular growth unless both *fhuA* and *fepA* were both removed.

Growth promotion in a Δ*fhuA*::FRT Δ*fepA*::*kan* pJL002 (FhuA complement) strain, however, suggests that the presence of enterobactin does not convert all Fc to its apo-form. Growth enhancement was also observed in a Δ*fhuA*::*kan* pKG116 strain (empty vector control) when only HCl was added, but the subsequent deletion of *fepA* resulted in no growth enhancement. Importantly, complementation of the double-knockout strain, Δ*fhuA*::FRT Δ*fepA*::*kan*, with pJL002 led to only minimal growth with the HCl negative control, but full growth enhancement with the addition of both HCl and Fc.

### Role of L8 in FhuA-dependent phage infection.

**FIG 7:**
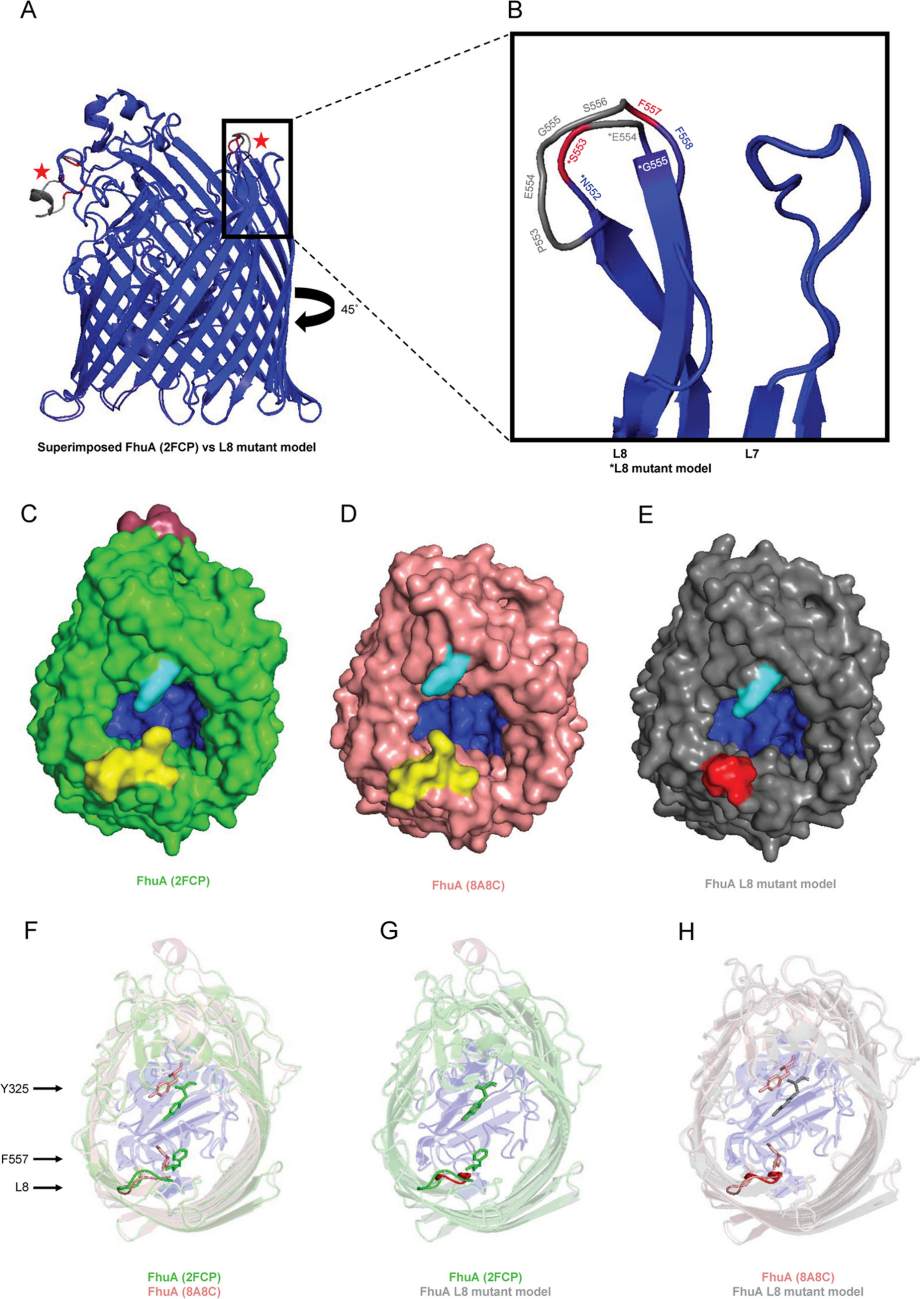
Comparisons of FhuA L8 placement in FhuA with and without T5 pb5 attachment and in a FhuA L8 mutant model. (A) Accuracy of the AlphaFold2 predicted structure of the FhuA L8 mutant was determined through superimposed root mean square deviation (RMSD) analysis against FhuA (2FCP). Regions of minimum (blue) and maximum (red) pairwise RMSD reveal sections of high and low similarity, respectively. Unaligned regions are marked in gray and the two regions showing high dissimilarity were in the 6×His region of FhuA (2FCP) and L8 (indicated by red stars). (B) Closeup of L8 with residues belonging to the mutant loop marked with an asterisk (*). (C to E) Top-down views of FhuA (2FCP) (C), FhuA (8A8C) (D), and the predicted L8 mutant structure (E) are shown as surface models and their β-barrels are colored green, salmon, and gray, respectively. The lumen (blue), wild-type L8 (yellow), mutant L8 (red), Y235 (cyan), and 6×His tag (raspberry) are also indicated. The structure of T5 pb5 is removed from 8A8C for clarity and allows observation of the shifts conferred by T5 pb5 attachment in FhuA L8 (F557) and L4 (Y325). (F to H) Shifts in L4 Y325 and L8 F557 positioning (shown as sticks) differ upon T5 attachment (F), but Y325 in the L8 mutant is predicted to coincide with Y325 placement in unbound FhuA (G) than in a phage-attached structure (H).

Removal of L8 resulted in complete loss of infection by SO1-like phages JLBYU37, JLBYU60, and Teewinot. This pattern coincides with previous studies that show that removal of L8 results in complete or significant reduction in phi80 or T5 phage infection, respectively (33). To further understand how mutations in the L8 mutant could confer phage resistance, we compared the AlphaFold2 predicted structure of our L8 mutant against the solved structures of FhuA with (8A8C) and without (2FCP) the T5 phage attachment protein pb5, which has recently been solved and published (51) (Fig. 7).

The plausibility of the AlphaFold2 prediction of the L8 mutant structure was assessed through superimposition onto FhuA (2FCP) and by calculating the root mean square deviation (RMSD, Fig. 7A) for the placement of the backbone C-alpha atoms. Only the regions that encode the 6×His tag of FhuA (2FCP) and the mutagenized section of L8 (Fig. 7B) resulted in a high maximum RMSD or low similarities between the two structures (indicated by the red stars in Fig. 7A). The top-down views of FhuA before (Fig. 7C) and after irreversible phage binding (Fig. 7D) are shown as surface model structures to show the residues exposed to the extracellular matrix. The structure of pb5 was removed from FhuA (8A8C) to aid in the visualization of L8 positioning and lumen exposure.

These comparisons highlight two residues, F557 (L8, yellow) and Y325 (which is in L4, cyan), which extend out into the entrance of the lumen and are shifted upon phage attachment. Replacement of residues DPEGSFF (D552-F558) with NSEG in the L8 mutant (Fig. 7E) results in a 3-amino acid reduction in loop length and a 56% reduction in solvent accessible surface area. This reduction results in a miniature L8 (shown in red) that is not predicted to stick out into the opening of the lumen. Superimposed structures of FhuA with and without phage attachment (Fig. 7F) clearly show a shift of Y325 and F557 placement (shown as stick structures) upon T5 attachment, but Y325 placement in the L8 mutant is expected to resemble FhuA structure prior to phage interaction (Fig. 7G) rather than post-T5 attachment (Fig. 7H).

Y325 and F557 predominance in the entrance of the lumen and shift upon T5 attachment suggests that these residues may also be involved in JLBYU37, JLBYU60, and Teewinot attachment. One observation is that these tyrosine and phenylalanine residues are hydrophobic amino acids that are fully exposed to the extracellular environment. Hydrophobic residues have been shown to play a supportive role in phage receptor binding (52), but whether they play a role in supporting initial RBP binding or serve as targets for irreversible phage attachment is still yet to be determined.

Removal of additional residues from L8 may also have a role in phage susceptibility. Analysis of the residues comprising L8 revealed the presence of two charged amino acids (D552 and E554) and two hydrophobic amino acids (F557 and F558). Both types of amino acids have been shown to play a role in ligand binding and may play an important role in tethering the phage to the cell. D552 and E554 have been predicted to play an important role in maintaining the rigidity of L8 (53), and collapse of L8 into the lumen of FhuA has been shown to confer T5 resistance (51). Removal of D552 may have adverse effects on the stability of L8 and alter its orientation on the cell surface. Consequently, this transition could confer complete phage resistance by blocking RBP access to the lumen of FhuA or preventing access to residues on L8 that are important for attachment. While the removal of D552 may cause L8 instability, we do not believe that the truncated version would be able to obstruct the lumen enough to prevent RBP access. Residues Y325 and F557 are both very susceptible to phage interaction, but removal of Y325 in the L4 mutant did not appear to impede JLBYU37, JLBYU60, and Teewinot infection.

Because of this, we believe that F557 may play an important role in infection by (i) initially tethering the phage to the cell surface, (ii) serving as an essential residue in conferring irreversible phage attachment, or (iii) triggering DNA ejection. We speculate that F557 may be involved in irreversible attachment or DNA ejection because the L8 mutation conferred complete phage resistance. However, this hypothesis was not tested in this study, and the mechanism behind L8-dependent phage resistance remains unknown.

### Utilization of the LPS heptose core in infection efficiency.

**FIG 8:**
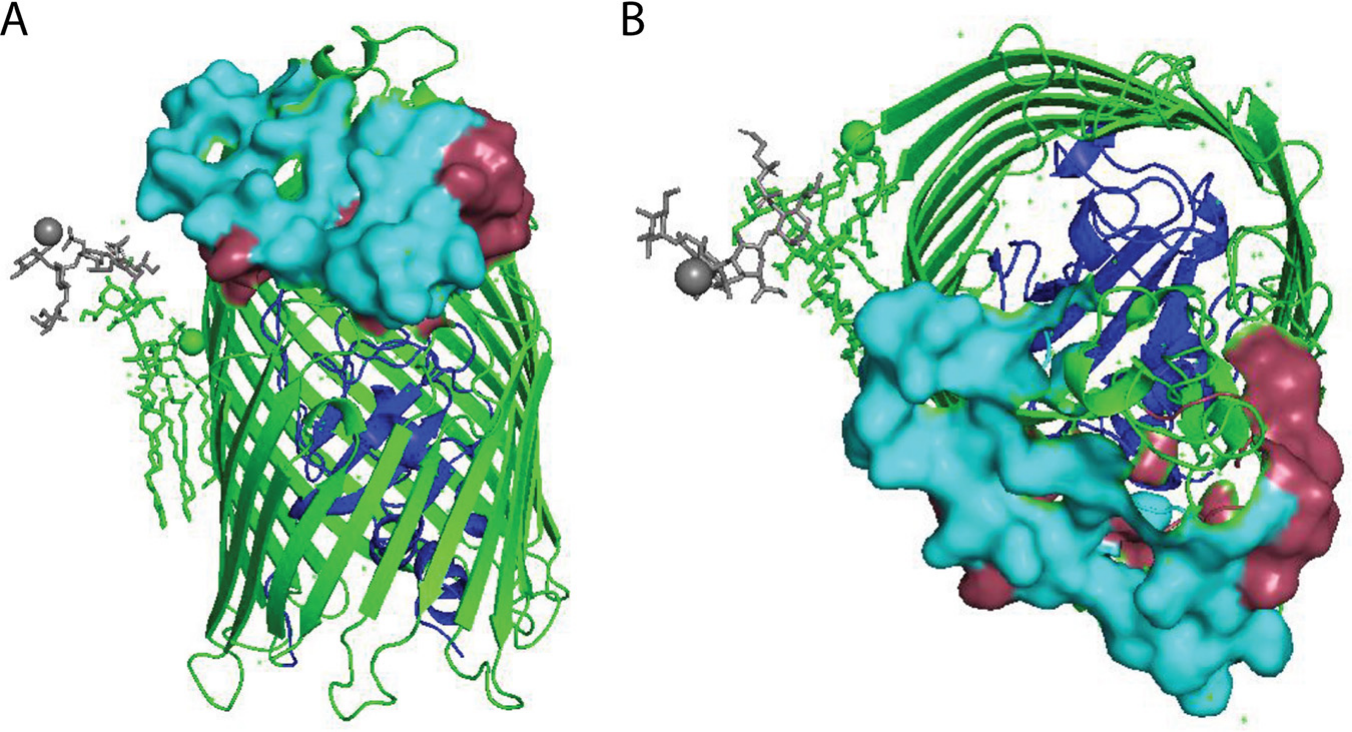
Role of LPS and FhuA loops in host recognition. Side (A) and top views (B) of FhuA (2FCP). Residues removed in the Δ*waaC* knockout are indicated in gray. FhuA is colored green and the cork, L3, and L5 are shown in blue, raspberry, and cyan, respectively. L3 and L5 are shown as surface models but FhuA is shown as a cartoon model.

SO1-like phages JLBYU37, JLBYU60, and Teewinot were affected by the L5 mutation, and significant reductions were observed upon the removal of the inner and outer core sugars of LPS. When observing the position of LPS relative to the placement of FhuA extracellular loops, L3 and L5 serve as a crown that partially wraps around the outside of the β-barrel (Fig. 8). LPS truncations resulting from the Δ*waaC* mutation are parallel to the base of L5 (Fig. 8A). Because of this, we propose that these phages utilize the sugars of LPS to bring them close to the surface of the cell and increase their probability of L5 discovery. Next, reversible binding of L5, which may serve as a ramp, leads directly to the lumen where irreversible binding can take place (Fig. 8B). Although a significant reduction was observed in the L3 mutant, this reduction remained the same upon LPS truncation. Consequently, we propose that L3 plays a role in L5 positioning but does not directly interact with phage tail fibers. L3 and L5 have been shown to be essential for Fc binding, whereas L3, L5, and L8 are important for Fc uptake (33). Interestingly, all 3 loops played various roles in phage attachment, so we propose that FhuA-dependent phages which target L5 have a selective advantage since this loop plays an important role in both Fc binding and transport.

The T1-like phage JLBYU41 was also affected by the L5 *fhuA* mutation, but the L8 deletion had no significant effect on infection until the LPS heptose core was removed. Interestingly, the truncation of LPS in the L5 mutant did not cause a greater reduction in JLBYU41 infection efficiency. Although LPS may play an important role in aiding in L8 discovery, complete resistance to JLBYU41 was only observed when multiple loops were deleted. Because of this, we propose that JLBYU41 infection requires a more complex interaction involving multiple extracellular loops and LPS.

### Evolution of FhuA loop preferences.

**FIG 9:**
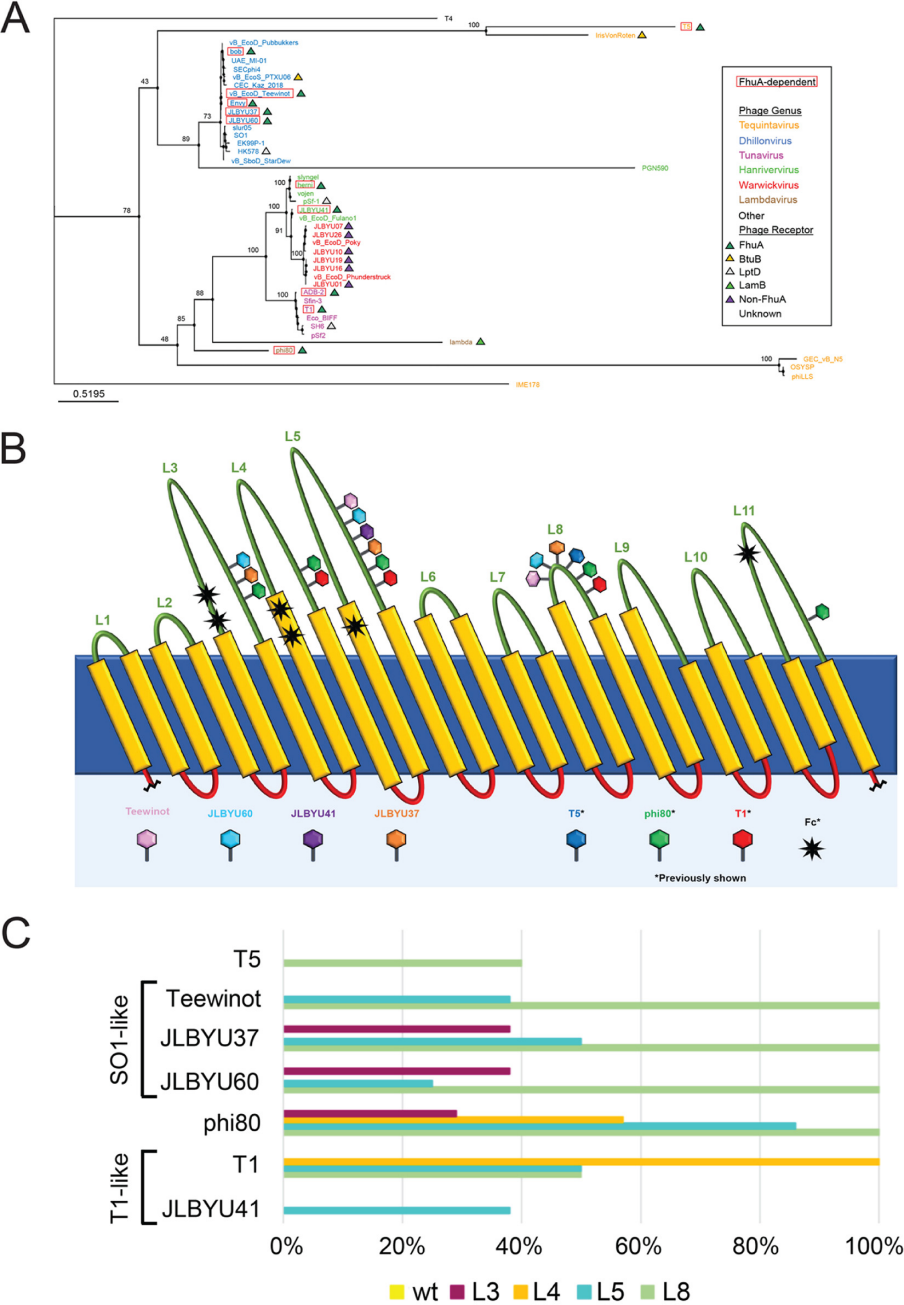
Evolutionary shift in FhuA loop preferences. (A) Evolutionary tree for the putative phage RBPs is colored based upon their respective genera: *Tequintavirus* (orange), *Dhillonvirus* (blue), *Tunavirus* (purple), *Hanrivervirus* (green), *Warwickvirus* (red), *Lambdavirus* (brown), and Other (black). Known phage receptors are indicated as FhuA (dark green triangle), BtuB (orange triangle), LptD (gray triangle), and LamB (light green triangle). Phages with unknown receptors do not have triangles; phages without a known receptor that were unaffected by a *fhuA* knockout are indicated by purple triangles. (B) Cartoon depiction of FhuA loops that were found to be important for FhuA-dependent phage attachment when the LPS heptose core was present. Loop deletions that caused a significant reduction (*P* < 0.005) in Teewinot (pink), JLBYU60 (light blue), JLBYU41 (purple), and JLBYU37 (orange) phage infection compared to the MG1655 Δ*fhuA*::FRT Δ*fepA*::*kan* pJL002 results are indicated by a phage icon on the corresponding loop. Results for T5 (blue), phi80 (green), and T1 (red) are based on data reported by Endriss et al. (33), and loops resulting in at least a 10^2^-fold reduction compared to infectivity on the wild-type strain were included. Loops involved in Fc binding (32, 33) are indicated by black stars. (C) Evolutionary shifts in FhuA loop preferences among various FhuA-dependent phages. Loop dependency is reported as the percent reduction that loop deletions caused in Teewinot, JLBYU37, JLBYU60, and JLBYU41 infection efficiency. Fold reductions for T5, T1, and phi80 were also based on results obtained by Endriss et al. (33); however, to maintain data comparability for this analysis, hazy spots were reported as being positive for phage infection.

Acquired dependence upon different regions of FhuA implies that various instances of positive selective pressure or homologous recombination may have occurred during the evolution of FhuA-dependent phages. To determine whether altered and conserved loop dependency across various phage families could be explained phylogenetically, we compared the evolutionary pathways of their predicted RBPs with their reported loop dependency (Fig. 9). The RBP evolutionary tree (Fig. 9A) represents phages from 7 different genera: *Tequatrovirus* (T4), *Tequintavirus* (orange), *Tunavirus* (purple), *Hanrivervirus* (green), *Warwickvirus* (red), *Dhillonvirus* (blue), and *Lambdavirus* (brown). The phages within these genera have *Siphoviridae* structure and contain at least one FhuA-dependent phage, except for T4 which has *Myoviridae* structure and is dependent upon OmpC and LPS (54). Although the targeted phage receptors are not known for every phage in the tree, those that have been experimentally determined target FhuA (13, 15, 21, 27–30, 39, 40, 55, 56), BtuB (cobalamin outer membrane transporter) (57), LptD (LPS biosynthesis) (32, 58), and LamB (maltose outer membrane transporter) (38, 56, 59), indicated by dark green, orange, light green, and gray triangles, respectively (Fig. 9A). Additional T1-like phages that were isolated in our lab (JLBYU07, JLBYU26, JLBYU10, JLBYU19, and JLBYU01) fall within the *Warwickvirus* genus and are included in this analysis due to their high similarity with JLBYU41. Although similar, these phages were not found to be FhuA-dependent and the receptors they target remain unknown (purple triangles).

Evolutionary analysis of predicted RBPs reveals an early split into two major clades, as discussed above. Briefly, the top clade contains the ancestor that gave rise to T5 and IrisVonRoten tequintaviruses, SO1-like *Dhillonvirus*, and T1-like *Hanrivervirus*. The bottom clade contains GEC_vB_N5, OSYSP, and phiLLS tequintaviruses, *Lambdavirus*, and T1-like *Tunavirus*, *Hanrivervirus*, and *Warwickvirus*. One possible reason for the divergence of these two clades is their dependence or lack of dependence on TonB in phage infection. T5 is known to be TonB-independent (21) (top major clade), whereas phi80 and T1 (bottom major clade) can only infect when both FhuA and TonB are present (15). In addition, the ancestor of T5 diverged from the ancestor of IrisVonRoten, which is a BtuB-dependent phage. Interestingly, BtuB and FhuA are both TonB-dependent transporters (60), and the only other identified BtuB-dependent phage is vB_EcoS_PTX06 (*Dhillonvirus* clade). Whether IrisVonRoten and vB_EcoS_PTX06 can infect independent of TonB expression, however, is unknown, and not enough information is available to conclude whether the generation of these two major clades occurred due to differences in TonB dependence in phage infection.

Dhillonviruses JLBYU37, JLBYU60, and Teewinot all required the presence of L8 (Fig. 9B) and fall within the same major clade as T5, which is also L8-dependent. Dhillonviruses bob and Envy were previously shown to be FhuA-dependent (56), but the loops they utilize in FhuA attachment were not assessed in this study. However, due to the predicted evolution of their RBPs, we propose that they may also be dependent upon L8.

Complete dependence upon L8 in the bottom major clade is only reported for *Lambdavirus* phi80 (33), which diverged early from the ancestor, giving rise to lambda and T1-like phages. However, as we move down the evolutionary path toward T1-like phages, T1 (*Tunavirus*) is found to be L4-dependent. This change could be explained by examining the roles the other loops play in FhuA attachment (Fig. 9C). Using this analysis, we find that phi80 is partially dependent upon L4 (33). While it is unknown whether L8 dependence was exchanged for L4 dependence in T1, these changes suggest that alterations in the host or environment may have made it advantageous for the ancestor that gave rise to T1 to become solely dependent upon L4. FhuA is an iron transporter which is expressed under iron-limited conditions (61). Because of this, changes in the amount of iron available in the environment during this time may have been a driving force for FhuA-dependent phage evolution.

JLBYU41, however, is not dependent upon L8 or any of the single loops analyzed in this study. This suggests that the selective pressure during its evolutionary history was either not as strong for single-loop dependence or that there was stronger selection to utilize more than one loop. These findings are consistent with the RBP dot-plot comparisons, which show high homology among all genera of T1-like phages except when the C-terminal regions of tunaviruses are involved. This suggests that RBP evolution among T1-like phages involved changes to the C terminus, which is also supported by the C-terminal RBP phylogenetic analysis.

### Conclusions.

Phage attachment onto a host is the primary step in the infection process and plays a major role in determining phage host range. Analysis of which FhuA regions contribute to FhuA-dependent phage infection revealed that the SO1-like phages JLBYU37, JLBYU60, and Teewinot are completely dependent upon FhuA L8. We hypothesize that mutations in L8 confer phage resistance by removing important residues, like F557, which may play an important role in initial binding or be directly involved in irreversible binding and DNA ejection. Complete resistance to T1-like JLBYU41 infection, however, was only conferred if multiple FhuA loops were altered. Removal of the LPS heptose core also significantly reduced phage infectivity, suggesting that these phages utilize LPS to guide them to very specific FhuA loops which serve as targets for reversible binding and guide the RBP to its irreversible binding site. Analysis of these FhuA-dependent phage binding profiles against the predicted evolutionary history of known FhuA-dependent phages and their close relatives revealed the conservation of L8 dependence among members of the *Tequintavirus*, *Dhillonvirus*, and *Lambdavirus* genera. However, L4 dependence in T1 and a lack of single-loop dependence in JLBYU41 infection was also observed within the ancestry of T1-like phages. Significant differences between the topology of the full-length and C-terminal RBP phylogenetic trees highlight the effect that positive selective pressure or homologous recombination may have had on FhuA-dependent RBP evolution. While the receptors targeted by each phage in the tree are not known, this comparison highlights possible instances of RBP evolution among FhuA-dependent phages. This study underscores the regions of FhuA that are targeted during FhuA-dependent phage attachment and the contribution of LPS to FhuA discovery, and begins to explore the evolutionary history which led to the diversity of FhuA-dependent RBPs.

## MATERIALS AND METHODS

### Phage isolation and receptor identification.

Coliphages were purified from raw sewage samples collected along the Wasatch front in Utah and spotted onto MG1655 E. coli mutants containing single-gene knockouts of known phage receptors. Single-deletion mutants were generated using Lambda Red recombination as previously described (62). Three FhuA-dependent phages, JLBYU37 (OK272488), JLBYU41 (OK272479), and JLBYU60 (OK272474), were identified using traditional plaque assays with 0.7% top agar (63), and complementation further revealed FhuA as the only phage receptor utilized by these phages. Phage scanning transmission electron microscope images were taken at the Brigham Young University (BYU) Microscopy Laboratory with a Helios NanoLab 600 dual-beam microscope, and phage samples were prepared for imaging by staining with either 2% phosphotungstate and bacitracin or uranyl acetate.

### Phage sequencing, genome assembly, and annotation.

Phage DNA was isolated using a Norgen Phage DNA Isolation kit (cat. no. 46800) and isolated phage DNA was sequenced using a NEBNext Ultra II FS DNA Library Prep kit (New England Biolabs, cat no. E7805S) and Illumina iSeq 100. Genomes were assembled with Geneious (64) version 8.1 using *de novo* assembly and medium-low sensitivity as previously described by Sharma et al. (65). All phages circularized upon *de novo* assembly and the coverage depths for each alignment were 40 to 172 (93 average) for JLBYU37, 13 to 147 (69.6 average) for JLBYU41, and 14 to 369 (175.7 average) for JLBYU60. DNA Master and GeneMark were used to determine open reading frames and each putative gene was annotated using BLASTp (66, 67) from NCBI.

### Genomic and structural comparisons.

Similar sequences for each phage were collected using BLASTp (66, 67) results that had an E value of ≥–07. Gepard (68) was used to perform whole-genome nucleotide comparisons and individual protein comparisons for putative phage MCP and RBPs.

### Generation of Δ*fhuA* loop deletion strains.

Mutant *fhuA* plasmids containing loop deletions were constructed using the NEB Q5 Site-Directed Mutagenesis kit (cat. no. E0554S). FhuA loops were replaced with a 4- or 5-amino acid sequence from loop 2 of OmpF as previously designed except for L3 (33). Each mutant plasmid was transformed into MG1655 Δ*fhuA*::FRT Δ*fepA*::*kan* strain to test for FhuA functionality and incorporation into the outer membrane with a Fc growth promotion test. Briefly, MG1655 Δ*fhuA*::FRT Δ*fepA*::*kan* mutant strains containing plasmids pJL004 to pJL009 (see Table S1) were tested for FhuA functionality and incorporation into the outer membrane by Fc growth promotion using M9 plates containing 100 μM dipyridyl (DIP) and 0.5 μM sodium salicylate. Next, 10 μM Fc was loaded with 10 μM FeCl_3_ by incubating the tubes at 37°C for 15 min. Next, 100 μL of ice-cold sterile double-distilled water (ddH_2_O) was added and spread over the outlined plates. A negative-control plate was also made by adding 10 mM HCl to 10 μM FeCl_3_ and preparing it as described above. Each strain was picked with a toothpick and patched first onto the FeCl_3_ + HCl negative Fc control plate and then onto the Fc + FeCl_3_ plate immediately afterward. Plates were pulled and read following a 24-h incubation at 37°C. Mutant plasmids were also transformed into a MG1655 Δ*fhuA*::FRT Δ*waaC*::*kan* strain to test the effects of the LPS inner and outer core on phage infection.

### Phage susceptibility spot test.

Spot tests were performed with 0.7% soft agar overlays using traditional methods. Briefly, overnight cultures of each mutant strain were normalized to the same optical density at 600 nm (OD_600_) reading and poured over a prewarmed plate in 4 mL 0.7% top agar. Once solidified, 5 μL of phage serial dilutions was spotted onto each mutant and incubated overnight at 37°C. An average of five replicates were performed for each phage and loop dependency was reported for mutants exhibiting significant fold reduction (*P* < 0.005) compared to the results for the MG1655 Δ*fhuA*::FRT Δ*fepA*::*kan* pJL002 or MG1655 Δ*fhuA*::FRT Δ*waaC*::*kan* pJL002 strain. Levels of significance were calculated using a Student’s *t* test with two-tailed distribution and two-sample equal variance. Hazy spots were scored positive for phage infection so that the fold reductions reported are due to the first dilution without a clearing or a hazy spot.

### Protein structural modeling.

Putative phage tail fiber-encoding genes were identified with BLASTp (66, 67) and the monomeric structure predictions for JLBYU37, JLBYU41, JLBYU60, and the FhuA L8 mutant were constructed using AlphaFold2 (69). Superimposition of unbound FhuA (2FCP), FhuA with T5 pb5 (8A8C) (51), and the predicted L8 mutant structures was performed in PyMOL (70) using the alignment plugin. A many-to-one super method alignment was performed using an outlier rejection with 5 cycles and a cutoff of 2.0. RMSD and solvent accessible surface area calculations and images were generated using the PyMOL scripts ‘ColorbyRMSD’ and ‘Get_area,’ respectively (71). Superimposition of structures of FhuA with T5 pb5 (8A8C) with the predicted structure of JLBYU37 gp22 was performed with the all-against-all structure comparison from Dali (72) and analyzed in PyMOL.

### Phage phylogenetic analysis.

Similar sequences for each phage MCP were obtained using BLASTp (66, 67) and the genome (nucleotide) and potential RBP (amino acid) sequences were collected from the same set of phages using the NCBI database. Multiple sequence alignments were generated with MAFFT (73) using the E-INS-i method (74), which compares conserved domains and accounts for the generalized affine gap cost (75). Model fit predictions (76) and maximum-likelihood tree generation (77) using the WAG+G4 model for the MCP tree and the LG+F+G4 model for substitution for the RBP tree were performed using IQ-TREE (78) with a resampling size of 1,000 and ultrafast bootstrap approximation (79). RBP C-terminal ends were analyzed using Jalview to show percent sequence identity among members of the same genus since little homology was observed when all sequences were compared. The start of the C-terminal end was chosen to be upstream of regions containing reduced percent identity in all genera. Tree generation was performed as outlined above using IQ-TREE and resulted in a tree using the VT+F+G4 model. Differences in tree topology between the full and C-terminal RBP trees were tested for significance using IQ-TREE topology evaluation. This showed significant differences in the Kishino-Hasegawa (80), Shimodaira-Hasegawa (81), and approximately unbiased tests (82) with 10,000 replicates.

### Data availability.

The genomes of the newly published phages JLBYU37 (accession no. OK272488), JLBYU41 (OK272479), and JLBYU60 (OK272474) are publicly available in the NCBI database.

## References

[1] Salmond GP, Fineran PC. 2015. A century of the phage: past, present and future. Nat Rev Microbiol 13:777–786. doi:10.1038/nrmicro3564. 26548913

[2] Wittebole X, De Roock S, Opal SM. 2014. A historical overview of bacteriophage therapy as an alternative to antibiotics for the treatment of bacterial pathogens. Virulence 5:226–235. doi:10.4161/viru.25991. 23973944 PMC3916379

[3] The L. 2022. Antimicrobial resistance: time to repurpose the Global Fund. Lancet 399:335. doi:10.1016/S0140-6736(22)00091-5. 35065769

[4] Chan BK, Turner PE, Kim S, Mojibian HR, Elefteriades JA, Narayan D. 2018. Phage treatment of an aortic graft infected with *Pseudomonas aeruginosa*. Evol Med Public Health 2018:60–66. doi:10.1093/emph/eoy005. 29588855 PMC5842392

[5] Dedrick RM, Guerrero-Bustamante CA, Garlena RA, Russell DA, Ford K, Harris K, Gilmour KC, Soothill J, Jacobs-Sera D, Schooley RT, Hatfull GF, Spencer H. 2019. Engineered bacteriophages for treatment of a patient with a disseminated drug-resistant *Mycobacterium abscessus*. Nat Med 25:730–733. doi:10.1038/s41591-019-0437-z. 31068712 PMC6557439

[6] Dedrick RM, Smith BE, Cristinziano M, Freeman KG, Jacobs-Sera D, Belessis Y, et al. 2022. Phage therapy of *Mycobacterium* infections: compassionate-use of phages in twenty patients with drug-resistant mycobacterial disease. Clin Infect Dis. 10.1093/cid/ciac453 PMC9825826 35676823

[7] Schooley RT, Biswas B, Gill JJ, Hernandez-Morales A, Lancaster J, Lessor L, Barr JJ, Reed SL, Rohwer F, Benler S, Segall AM, Taplitz R, Smith DM, Kerr K, Kumaraswamy M, Nizet V, Lin L, McCauley MD, Strathdee SA, Benson CA, Pope RK, Leroux BM, Picel AC, Mateczun AJ, Cilwa KE, Regeimbal JM, Estrella LA, Wolfe DM, Henry MS, Quinones J, Salka S, Bishop-Lilly KA, Young R, Hamilton T. 2017. Development and use of personalized bacteriophage-based therapeutic cocktails to treat a patient with a disseminated resistant Acinetobacter baumannii infection. Antimicrob Agents Chemother 61. doi:10.1128/AAC.00954-17. PMC5610518 28807909

[8] Rao DN, Dryden DT, Bheemanaik S. 2014. Type III restriction-modification enzymes: a historical perspective. Nucleic Acids Res 42:45–55. doi:10.1093/nar/gkt616. 23863841 PMC3874151

[9] Westra ER, Swarts DC, Staals RH, Jore MM, Brouns SJ, van der Oost J. 2012. The CRISPRs, they are a-changin’: how prokaryotes generate adaptive immunity. Annu Rev Genet 46:311–339. doi:10.1146/annurev-genet-110711-155447. 23145983

[10] Lopatina A, Tal N, Sorek R. 2020. Abortive infection: bacterial suicide as an antiviral immune strategy. Annu Rev Virol 7:371–384. doi:10.1146/annurev-virology-011620-040628. 32559405

[11] Wang Y, Fan H, Tong Y. 2023. Unveil the secret of the bacteria and phage arms race. Int J Mol Sci 24. doi:10.3390/ijms24054363. PMC10002423 36901793

[12] Duckworth DH (ed), History and basic properties of bacterial viruses 1987.

[13] Bertozzi Silva J, Storms Z, Sauvageau D. 2016. Host receptors for bacteriophage adsorption. FEMS Microbiology Lett 363:fnw002. doi:10.1093/femsle/fnw002. 26755501

[14] Chatterjee S, Rothenberg E. 2012. Interaction of bacteriophage l with its *E. coli* receptor, LamB. Viruses 4:3162–3178. doi:10.3390/v4113162. 23202520 PMC3509688

[15] Garen A, Puck TT. 1951. The first two steps of the invasion of host cells by bacterial viruses. II. J Exp Med 94:177–189. doi:10.1084/jem.94.3.177. 14861377 PMC2136106

[16] Hagge SO, de Cock H, Gutsmann T, Beckers F, Seydel U, Wiese A. 2002. Pore formation and function of phosphoporin PhoE of *Escherichia coli* are determined by the core sugar moiety of lipopolysaccharide. J Biol Chem 277:34247–34253. doi:10.1074/jbc.M201950200. 12091383

[17] Washizaki A, Yonesaki T, Otsuka Y. 2016. Characterization of the interactions betweenEscherichia colireceptors, LPS and OmpC, and bacteriophage T4 long tail fibers. Microbiologyopen 5:1003–1015. doi:10.1002/mbo3.384. 27273222 PMC5221442

[18] Riede I. 1987. Receptor specificity of the short tail fibres (gp12) of T-even type *Escherichia coli* phages. Mol Gen Genet 206:110–115. doi:10.1007/BF00326544. 3553859

[19] Yehl K, Lemire S, Yang AC, Ando H, Mimee M, Der Torossian Torres M. 2019. Engineering phage host-range and suppressing bacterial resistance through phage tail fiber mutagenesis. 10.1016/j.cell.2019.09.015 PMC6924272 31585083

[20] Black PN. 1988. The fadL gene product of *Escherichia coli* is an outer membrane protein required for uptake of long-chain fatty acids and involved in sensitivity to bacteriophage T2. J Bacteriol 170:2850–2854. doi:10.1128/jb.170.6.2850-2854.1988. 3286621 PMC211212

[21] Hantke K, Braun V. 1978. Functional interaction of the *tonA*/*tonB* receptor system in *Escherichia coli*. J Bacteriol 135:190–197. doi:10.1128/jb.135.1.190-197.1978. 353030 PMC224805

[22] Beher MG, Pugsley AP. 1981. Coliphage which requires either the LamB protein or the OmpC protein for adsorption to *Escherichia coli* K-12. J Virol 38:372–375. doi:10.1128/JVI.38.1.372-375.1981. 6454006 PMC171160

[23] Braun V, Hantke K. 2011. Recent insights into iron import by bacteria. Curr Opin Chem Biol 15:328–334. doi:10.1016/j.cbpa.2011.01.005. 21277822

[24] Krewulak KD, Vogel HJ. 2011. TonB or not TonB: is that the question? Biochem Cell Biol 89:87–97. doi:10.1139/o10-141. 21455261

[25] Nairz M, Schroll A, Sonnweber T, Weiss G. 2010. The struggle for iron: a metal at the host-pathogen interface. Cell Microbiol 12:1691–1702. doi:10.1111/j.1462-5822.2010.01529.x. 20964797

[26] Skaar EP. 2010. The battle for iron between bacterial pathogens and their vertebrate hosts. PLoS Pathog 6:e1000949. doi:10.1371/journal.ppat.1000949. 20711357 PMC2920840

[27] Braun V, Schaller K, Wolff H. 1973. A common receptor protein for phage T5 and colicin M in the outer membrane of *Escherichia coli* B. Biochim Biophys Acta 323:87–97. doi:10.1016/0005-2736(73)90433-1. 4584483

[28] Braun V, Wolff H. 1973. Characterization of the receptor protein for phage T5 and colicin M in the outer membrane of *E. coli* B. FEBS Lett 34:77–80. doi:10.1016/0014-5793(73)80707-0. 4580999

[29] Hancock RW, Braun V. 1976. Nature of the energy requirement for the irreversible adsorption of bacteriophages T1 and phi80 to *Escherichia coli*. J Bacteriol 125:409–415. doi:10.1128/jb.125.2.409-415.1976. 128553 PMC236097

[30] Wayne R, Neilands JB. 1975. Evidence for common binding sites for ferrichrome compounds and bacteriophage phi 80 in the cell envelope of *Escherichia coli*. J Bacteriol 121:497–503. doi:10.1128/jb.121.2.497-503.1975. 803957 PMC245958

[31] Grinter R, Lithgow T. 2019. The structure of the bacterial iron-catecholate transporter Fiu suggests that it imports substrates via a two-step mechanism. J Biol Chem 294:19523–19534. doi:10.1074/jbc.RA119.011018. 31712312 PMC6926462

[32] Braun V, Braun M. 2002. Active transport of iron and siderophore antibiotics. Curr Opin Microbiol 5:194–201. doi:10.1016/s1369-5274(02)00298-9. 11934617

[33] Endriss F, Braun V. 2004. Loop deletions indicate regions important for FhuA transport and receptor functions in *Escherichia coli*. J Bacteriol 186:4818–4823. doi:10.1128/JB.186.14.4818-4823.2004. 15231815 PMC438571

[34] Ferguson AD, Braun V, Fiedler HP, Coulton JW, Diederichs K, Welte W. 2000. Crystal structure of the antibiotic albomycin in complex with the outer membrane transporter FhuA. Protein Sci 9:956–963. doi:10.1110/ps.9.5.956. 10850805 PMC2144648

[35] Pugsley AP, Zimmerman W, Wehrli W. 1987. Highly efficient uptake of a rifamycin derivative via the FhuA-TonB-dependent uptake route in *Escherichia coli*. J Gen Microbiol 133:3505–3511. doi:10.1099/00221287-133-12-3505. 3332686

[36] Ferguson AD, Ködding J, Walker G, Bös C, Coulton JW, Diederichs K, Braun V, Welte W. 2001. Active transport of an antibiotic rifamycin derivative by the outer-membrane protein FhuA. Structure 9:707–716. doi:10.1016/s0969-2126(01)00631-1. 11587645

[37] Salomon RA, Farias RN. 1993. The FhuA protein is involved in microcin 25 uptake. J Bacteriol 175:7741–7742. doi:10.1128/jb.175.23.7741-7742.1993. 8244949 PMC206939

[38] Randall-Hazelbauer LL, Schwartz M. 1973. Isolation of the bacteriophage lambda receptor from *Escherichia coli*. J Bacteriol 116:1436–1446. doi:10.1128/jb.116.3.1436-1446.1973. 4201774 PMC246503

[39] Henning U, Jann K. 1979. Two-component nature of bacteriophage T4 receptor activity in *Escherichia coli* K-12. J Bacteriol 137:664–666. doi:10.1128/jb.137.1.664-666.1979. 368036 PMC218498

[40] Tamaki S, Sato T, Matsuhashi M. 1971. Role of lipopolysaccharides in antibiotic resistance and bacteriophage adsorption of *Escherichia coli* K-12. J Bacteriol 105:968–975. doi:10.1128/jb.105.3.968-975.1971. 4926688 PMC248525

[41] Turner D, Shkoporov AN, Lood C, Millard AD, Dutilh BE, Alfenas-Zerbini P, van Zyl LJ, Aziz RK, Oksanen HM, Poranen MM, Kropinski AM, Barylski J, Brister JR, Chanisvili N, Edwards RA, Enault F, Gillis A, Knezevic P, Krupovic M, Kurtböke I, Kushkina A, Lavigne R, Lehman S, Lobocka M, Moraru C, Moreno Switt A, Morozova V, Nakavuma J, Reyes Muñoz A, Rūmnieks J, Sarkar BL, Sullivan MB, Uchiyama J, Wittmann J, Yigang T, Adriaenssens EM. 2023. Abolishment of morphology-based taxa and change to binomial species names: 2022 taxonomy update of the ICTV bacterial viruses subcommittee. Arch Virol 168:74. doi:10.1007/s00705-022-05694-2. 36683075 PMC9868039

[42] Schoch CL, Ciufo S, Domrachev M, Hotton CL, Kannan S, Khovanskaya R, Leipe D, Mcveigh R, O’Neill K, Robbertse B, Sharma S, Soussov V, Sullivan JP, Sun L, Turner S, Karsch-Mizrachi I. 2020. NCBI Taxonomy: a comprehensive update on curation, resources and tools. Database (Oxford) 2020:baaa062. doi:10.1093/database/baaa062. 32761142 PMC7408187

[43] Dion MB, Oechslin F, Moineau S. 2020. Phage diversity, genomics and phylogeny. Nat Rev Microbiol 18:125–138. doi:10.1038/s41579-019-0311-5. 32015529

[44] Purnell MG, Andersen K, Bell A, Briscoe JT, Brown HMF, Carr EL, Doney J, Folsom PF, Green C, Harris EH, Huhem E, Jensen RM, Johnson L, Jones C, Lambert AS, Loertscher E, Newey CR, Porter M, Rallison J, Sharma R, Sork C, Soule S, Stewart JB, Stoker T, Tayler S, Thompson DW, Thurgood TL, Walker J, Breakwell DP, Casjens SR, Grose JH. 2022. Complete genome sequences of five SO-1-like *Siphoviridae* bacteriophages that infect *Enterobacteriales*. Microbiol Resour Announc 11:e0122421. doi:10.1128/mra.01224-21. 35293823 PMC9022532

[45] Latka A, Leiman PG, Drulis-Kawa Z, Briers Y. 2019. Modeling the architecture of depolymerase-containing receptor binding proteins in *Klebsiella* phages. Front Microbiol 10:2649. doi:10.3389/fmicb.2019.02649. 31803168 PMC6872550

[46] Haggard-Ljungquist E, Halling C, Calendar R. 1992. DNA sequences of the tail fiber genes of bacteriophage P2: evidence for horizontal transfer of tail fiber genes among unrelated bacteriophages. J Bacteriol 174:1462–1477. doi:10.1128/jb.174.5.1462-1477.1992. 1531648 PMC206541

[47] Legrand P, Collins B, Blangy S, Murphy J, Spinelli S, Gutierrez C, Richet N, Kellenberger C, Desmyter A, Mahony J, van Sinderen D, Cambillau C. 2016. The atomic structure of the phage Tuc2009 baseplate tripod suggests that host recognition involves two different carbohydrate binding modules. mBio 7:e01781-15. doi:10.1128/mBio.01781-15. 26814179 PMC4742702

[48] Spinelli S, Campanacci V, Blangy S, Moineau S, Tegoni M, Cambillau C. 2006. Modular structure of the receptor binding proteins of Lactococcus lactis phages. The RBP structure of the temperate phage TP901-1. J Biol Chem 281:14256–14262. doi:10.1074/jbc.M600666200. 16549427

[49] Tremblay DM, Tegoni M, Spinelli S, Campanacci V, Blangy S, Huyghe C, Desmyter A, Labrie S, Moineau S, Cambillau C. 2006. Receptor-binding protein of *Lactococcus lactis* phages: identification and characterization of the saccharide receptor-binding site. J Bacteriol 188:2400–2410. doi:10.1128/JB.188.7.2400-2410.2006. 16547026 PMC1428394

[50] Li GW, Burkhardt D, Gross C, Weissman JS. 2014. Quantifying absolute protein synthesis rates reveals principles underlying allocation of cellular resources. Cell 157:624–635. doi:10.1016/j.cell.2014.02.033. 24766808 PMC4006352

[51] van den Berg B, Silale A, Basle A, Brandner AF, Mader SL, Khalid S. 2022. Structural basis for host recognition and superinfection exclusion by bacteriophage T5. Proc Natl Acad Sci USA 119:e2211672119. doi:10.1073/pnas.2211672119. 36215462 PMC9586334

[52] Meng R, Jiang M, Cui Z, Chang J-Y, Yang K, Jakana J, Yu X, Wang Z, Hu B, Zhang J. 2019. Structural basis for the adsorption of a single-stranded RNA bacteriophage. Nat Commun 10:3130. doi:10.1038/s41467-019-11126-8. 31311931 PMC6635492

[53] Mohammad MM, Movileanu L. 2010. Impact of distant charge reversals within a robust beta-barrel protein pore. J Phys Chem B 114:8750–8759. doi:10.1021/jp101311s. 20540583 PMC2907733

[54] Yu F, Mizushima S. 1982. Roles of lipopolysaccharide and outer membrane protein OmpC of *Escherichia coli* K-12 in the receptor function for bacteriophage T4. J Bacteriol 151:718–722. doi:10.1128/jb.151.2.718-722.1982. 7047495 PMC220313

[55] Hantke K. 2020. Compilation of *Escherichia coli* K-12 outer membrane phage receptors: their function and some historical remarks. FEMS Microbiology Lett 367:fnaa013. doi:10.1093/femsle/fnaa013. 32009155

[56] Maffei E, Shaidullina A, Burkolter M, Heyer Y, Estermann F, Druelle V, Sauer P, Willi L, Michaelis S, Hilbi H, Thaler DS, Harms A. 2021. Systematic exploration of *Escherichia coli* phage-host interactions with the BASEL phage collection. PLoS Biol 19:e3001424. doi:10.1371/journal.pbio.3001424. 34784345 PMC8594841

[57] Di Girolamo PM, Bradbeer C. 1971. Transport of vitamin B 12 in *Escherichia coli*. J Bacteriol 106:745–750. doi:10.1128/jb.106.3.745-750.1971. 4934062 PMC248688

[58] Wu T, McCandlish AC, Gronenberg LS, Chng SS, Silhavy TJ, Kahne D. 2006. Identification of a protein complex that assembles lipopolysaccharide in the outer membrane of *Escherichia coli*. Proc Natl Acad Sci USA 103:11754–11759. doi:10.1073/pnas.0604744103. 16861298 PMC1544242

[59] Wang YF, Dutzler R, Rizkallah PJ, Rosenbusch JP, Schirmer T. 1997. Channel specificity: structural basis for sugar discrimination and differential flux rates in maltoporin. J Mol Biol 272:56–63. doi:10.1006/jmbi.1997.1224. 9299337

[60] Reynolds PR, Mottur GP, Bradbeer C. 1980. Transport of vitamin B12 in *Escherichia coli*. Some observations on the roles of the gene products of BtuC and TonB. J Biol Chem 255:4313–4319. doi:10.1016/S0021-9258(19)85667-3. 6768753

[61] Klebba PE, McIntosh MA, Neilands JB. 1982. Kinetics of biosynthesis of iron-regulated membrane proteins in *Escherichia coli*. J Bacteriol 149:880–888. doi:10.1128/jb.149.3.880-888.1982. 6174499 PMC216474

[62] Datsenko KA, Wanner BL. 2000. One-step inactivation of chromosomal genes in *Escherichia coli* K-12 using PCR products. Proc Natl Acad Sci USA 97:6640–6645. doi:10.1073/pnas.120163297. 10829079 PMC18686

[63] Kropinski AM, Mazzocco A, Waddell TE, Lingohr E, Johnson RP. 2009. Enumeration of bacteriophages by double agar overlay plaque assay. Methods Mol Biol 501:69–76. doi:10.1007/978-1-60327-164-6_7. 19066811

[64] Kearse M, Moir R, Wilson A, Stones-Havas S, Cheung M, Sturrock S, Buxton S, Cooper A, Markowitz S, Duran C, Thierer T, Ashton B, Meintjes P, Drummond A. 2012. Geneious Basic: an integrated and extendable desktop software platform for the organization and analysis of sequence data. Bioinformatics 28:1647–1649. doi:10.1093/bioinformatics/bts199. 22543367 PMC3371832

[65] Sharma R, Berg JA, Beatty NJ, Choi MC, Cowger AE, Cozzens BJR, Duncan SG, Fajardo CP, Ferguson HP, Galbraith T, Herring JA, Hoj TR, Durrant JL, Hyde JR, Jensen GL, Ke SY, Killpack S, Kruger JL, Lawrence EEK, Nwosu IO, Tam TC, Thompson DW, Tueller JA, Ward MEH, Webb CJ, Wood ME, Yeates EL, Baltrus DA, Breakwell DP, Hope S, Grose JH. 2018. Genome sequences of nine *Erwinia amylovora* bacteriophages. Microbiol Resour Announc 7:e0094-18. doi:10.1128/MRA.00944-18. PMC6256631 30533701

[66] Boratyn GM, Camacho C, Cooper PS, Coulouris G, Fong A, Ma N, Madden TL, Matten WT, McGinnis SD, Merezhuk Y, Raytselis Y, Sayers EW, Tao T, Ye J, Zaretskaya I. 2013. BLAST: a more efficient report with usability improvements. Nucleic Acids Res 41:W29–33. doi:10.1093/nar/gkt282. 23609542 PMC3692093

[67] Madden TL, Tatusov RL, Zhang J. 1996. Applications of network BLAST server. Methods Enzymol 266:131–141. doi:10.1016/s0076-6879(96)66011-x. 8743682

[68] Krumsiek J, Arnold R, Rattei T. 2007. Gepard: a rapid and sensitive tool for creating dotplots on genome scale. Bioinformatics 23:1026–1028. doi:10.1093/bioinformatics/btm039. 17309896

[69] Jumper J, Evans R, Pritzel A, Green T, Figurnov M, Ronneberger O, Tunyasuvunakool K, Bates R, Žídek A, Potapenko A, Bridgland A, Meyer C, Kohl SAA, Ballard AJ, Cowie A, Romera-Paredes B, Nikolov S, Jain R, Adler J, Back T, Petersen S, Reiman D, Clancy E, Zielinski M, Steinegger M, Pacholska M, Berghammer T, Bodenstein S, Silver D, Vinyals O, Senior AW, Kavukcuoglu K, Kohli P, Hassabis D. 2021. Highly accurate protein structure prediction with AlphaFold. Nature 596:583–589. doi:10.1038/s41586-021-03819-2. 34265844 PMC8371605

[70] Delano WL (ed), The PyMOL molecular graphics system 2002.

[71] Mooers BHM. 2020. Shortcuts for faster image creation in PyMOL. Protein Sci 29:268–276. doi:10.1002/pro.3781. 31710740 PMC6933860

[72] Holm L, Rosenstrom P. 2010. Dali server: conservation mapping in 3D. Nucleic Acids Res 38:W545–9. doi:10.1093/nar/gkq366. 20457744 PMC2896194

[73] Katoh K, Misawa K, Kuma K, Miyata T. 2002. MAFFT: a novel method for rapid multiple sequence alignment based on fast Fourier transform. Nucleic Acids Res 30:3059–3066. doi:10.1093/nar/gkf436. 12136088 PMC135756

[74] Katoh K, Toh H. 2008. Recent developments in the MAFFT multiple sequence alignment program. Brief Bioinform 9:286–298. doi:10.1093/bib/bbn013. 18372315

[75] Altschul SF. 1998. Generalized affine gap costs for protein sequence alignment. Proteins 32:88–96. doi:10.1002/(SICI)1097-0134(19980701)32:1<88::AID-PROT10>3.0.CO;2-J. 9672045

[76] Kalyaanamoorthy S, Minh BQ, Wong TKF, von Haeseler A, Jermiin LS. 2017. ModelFinder: fast model selection for accurate phylogenetic estimates. Nat Methods 14:587–589. doi:10.1038/nmeth.4285. 28481363 PMC5453245

[77] Trifinopoulos J, Nguyen LT, von Haeseler A, Minh BQ. 2016. W-IQ-TREE: a fast online phylogenetic tool for maximum likelihood analysis. Nucleic Acids Res 44:W232–W235. doi:10.1093/nar/gkw256. 27084950 PMC4987875

[78] Nguyen LT, Schmidt HA, von Haeseler A, Minh BQ. 2015. IQ-TREE: a fast and effective stochastic algorithm for estimating maximum-likelihood phylogenies. Mol Biol Evol 32:268–274. doi:10.1093/molbev/msu300. 25371430 PMC4271533

[79] Hoang DT, Chernomor O, von Haeseler A, Minh BQ, Vinh LS. 2018. UFBoot2: improving the ultrafast bootstrap approximation. Mol Biol Evol 35:518–522. doi:10.1093/molbev/msx281. 29077904 PMC5850222

[80] Kishino H, Hasegawa M. 1989. Evaluation of the maximum likelihood estimate of the evolutionary tree topologies from DNA sequence data, and the branching order in hominoidea. J Mol Evol 29:170–179. doi:10.1007/BF02100115. 2509717

[81] Shimodaira H, Hasegawa M. 1999. Multiple comparisons of log-likelihoods with applications to phylogenetic inference. Mol Biol Evol 16:1114–1116. doi:10.1093/oxfordjournals.molbev.a026201.

[82] Shimodaira H. 2002. An approximately unbiased test of phylogenetic tree selection. Syst Biol 51:492–508. doi:10.1080/10635150290069913. 12079646

[83] Makalatia K, Kakabadze E, Wagemans J, Grdzelishvili N, Bakuradze N, Natroshvili G, et al. 2020. Characterization of *Salmonella* isolates from various geographical regions of the Caucasus and their susceptibility to bacteriophages. Viruses 12:1418. doi:10.3390/v12121418. 33321823 PMC7764154

[84] Yesil M, Huang E, Yang X, Yousef A. 2017. Complete genome sequence of *Escherichia* phage OSYSP. Genome Announcements 5:e00880-17. doi:10.1128/genomeA.00880-17. 29051235 PMC5646388

[85] Amarillas L, Rubi-Rangel L, Chaidez C, Gonzalez-Robles A, Lightbourn-Rojas L, Leon-Felix J. 2017. Isolation and characterization of phiLLS, a novel phage with potential biocontrol agent against multidrug-resistant *Escherichia coli*. Front Microbiol 8:1355. doi:10.3389/fmicb.2017.01355. 28785246 PMC5519627

[86] Li F, Li L, Na S, Zhao J, Liu F, Liu P, Li Y, Li M, Lei M, Zhang D, Nazir A, Wang G. 2023. Isolation, characterization and genomic analysis of a novel phage IME178 with lytic activity against *Escherichia coli*. Microb Pathog 179:106099. doi:10.1016/j.micpath.2023.106099. 37060965

[87] Olsen NS, Forero-Junco L, Kot W, Hansen LH. 2020. Exploring the remarkable diversity of culturable *Escherichia coli* phages in the Danish wastewater environment. Viruses 12:986. doi:10.3390/v12090986. 32899836 PMC7552041

[88] Sultan-Alolama MI, El-Tarabily KA, Vijayan R. 2021. Complete genome sequence of *Escherichia coli* O157:H7 phage UAE_MI-01, Isolated from bird feces. Microbiol Resour Announc 10:e0034821. doi:10.1128/MRA.00348-21. 34264095 PMC8281071

[89] Millman A, Bernheim A, Stokar-Avihail A, Fedorenko T, Voichek M, Leavitt A, Oppenheimer-Shaanan Y, Sorek R. 2020. Bacterial retrons function in anti-phage defense. Cell 183:1551–1561.e12. doi:10.1016/j.cell.2020.09.065. 33157039

[90] Korf IHE, Meier-Kolthoff JP, Adriaenssens EM, Kropinski AM, Nimtz M, Rohde M, van Raaij MJ, Wittmann J. 2019. Still something to discover: novel insights into *Escherichia coli* phage diversity and taxonomy. Viruses 11:454. doi:10.3390/v11050454. 31109012 PMC6563267

[91] Moldakhanov YS, Alexyuk MS, Bogoyavlenskiy AP, Alexyuk PG, Turmagambetova AS, Zaitseva IA, Sokolova NS, Akanova KS, Anarkulova EI, Omirtaeva ES, Berezin VE. 2019. Complete genome sequence of *Escherichia*-infecting phage CEC_KAZ_2018, isolated from soil. Microbiol Resour Announc 8:e00540-19. doi:10.1128/MRA.00540-19. 31488525 PMC6728635

[92] Malki K, Sible E, Cooper A, Garretto A, Bruder K, Watkins SC, Putonti C. 2016. Seven bacteriophages isolated from the female urinary microbiota. Genome Announc 4:e01003-16. doi:10.1128/genomeA.01003-16. 27881533 PMC5122675

[93] Kim N, Gu MJ, Kye Y-C, Ju Y-J, Hong R, Ju DB, Pyung YJ, Han SH, Park B-C, Yun C-H. 2022. Bacteriophage EK99P-1 alleviates enterotoxigenic *Escherichia coli* K99-induced barrier dysfunction and inflammation. Sci Rep 12:941. doi:10.1038/s41598-022-04861-4. 35042907 PMC8766502

[94] Smith R, O’Hara M, Hobman JL, Millard AD. 2015. Draft genome sequences of 14 *Escherichia coli* phages isolated from cattle slurry. Genome Announc 3:e01364-15. doi:10.1128/genomeA.01364-15. 26722010 PMC4698387

[95] Aizawa S, Matsushiro A. 1975. Studies on temperature sensitive growth of phage phi 80. I. Prophage excision. Virology 67:168–178. doi:10.1016/0042-6822(75)90414-6. 1099782

[96] Rotman E, Kouzminova E, Plunkett G, 3rd, Kuzminov A. 2012. Genome of Enterobacteriophage Lula/phi80 and insights into its ability to spread in the laboratory environment. J Bacteriol 194:6802–6817. doi:10.1128/JB.01353-12. 23042999 PMC3510586

[97] Roberts MD, Martin NL, Kropinski AM. 2004. The genome and proteome of coliphage T1. Virology 318:245–266. doi:10.1016/j.virol.2003.09.020. 14972552

[98] Jun JW, Kim HJ, Yun SK, Chai JY, Lee BC, Park SC. 2016. Isolation and comparative genomic analysis of T1-like *Shigella* bacteriophage pSf-2. Curr Microbiol 72:235–241. doi:10.1007/s00284-015-0935-2. 26612033

[99] Hamdi S, Rousseau GM, Labrie SJ, Tremblay DM, Kourda RS, Ben Slama K, Moineau S. 2017. Characterization of two polyvalent phages infecting *Enterobacteriaceae*. Sci Rep 7:40349. doi:10.1038/srep40349. 28091598 PMC5238451

[100] Bhensdadia DV, Bhimani HD, Rawal CM, Kothari VV, Raval VH, Kothari CR, Patel AB, Bhatt VD, Parmar NR, Sajnani MR, Koringa PG, Joshi CG, Singh SP, Kothari RK. 2013. Complete genome sequence of *Escherichia* phage ADB-2 isolated from a fecal sample of poultry. Genome Announc 1:e0004313. doi:10.1128/genomeA.00043-13. 23516186 PMC3622963

[101] Jun JW, Kim JH, Shin SP, Han JE, Chai JY, Park SC. 2013. Characterization and complete genome sequence of the *Shigella* bacteriophage pSf-1. Res Microbiol 164:979–986. doi:10.1016/j.resmic.2013.08.007. 24012542

[102] Cranston A, Danielson P, Arens DK, Barker A, Birch EK, Brown H, Carr E, Cero P, Chow J, Correa E, Dean J, Dunn M, Eberhard N, Egbert A, Foster K, Gaertner R, Gleave A, Gomez A, Gordon JB, Harris EB, Heaps C, Hyer M, Johnson A, Johnson L, Kim M, Kruger JL, Leonard T, LeSueur A, Lima S, Marshall N, Moulton R, Newey CR, Owen D, Packard A, Rolfson A, Suorsa AR, Rodriguez W, Sandoval C, Sharma R, Smith A, Sork C, Soule C, Soule S, Stewart J, Stoker T, Thompson DW, Thurgood T, Walker J, Zaugg E, Casjens SR, et al. 2022. Genome sequences of 22 T1-like bacteriophages that infect *Enterobacteriales*. Microbiol Resour Announc 11:e0122121. doi:10.1128/mra.01221-21. 35389258 PMC9119101

