# Supplementary material for: Characterization of the Attachment of Three New Coliphages onto the Ferrichrome Transporter FhuA
Source: J Virol. 2023 Jun 13;97(7):e00667-23. doi: 10.1128/jvi.00667-23 (PMC10373560; doi:10.1128/jvi.00667-23)
Supplement: Supplemental file 1 — Fig. S1 and Tables S1 to S3. Download jvi.00667-23-s0001.pdf, PDF file, 0.2 MB [file jvi.00667-23-s0001.pdf]

Supplementary Material

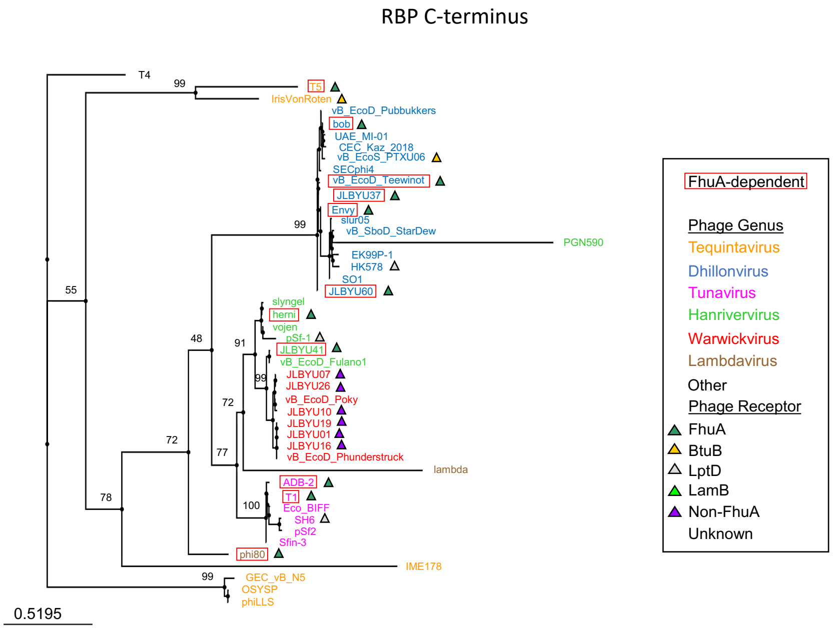

Fig S1 Evolutionary analysis of the C-terminal region of FhuA-dependent phage RBPs and their homologs. The evolutionary tree of C-terminal RBPs was constructed by removing 400-803 amino acids from the N-terminal region of the predicted phage RBPs (see Supplementary Table S3). Known FhuA-dependent phages are outlined with a red box and Tequintavirus (orange), Dhillonvirus (blue), Tunavirus (purple), Hanrivervirus (green), Warwickvirus (red) and Lambdavirus (brown) are distinguished. Information regarding the receptors a phage targets is marked with green, gold, gray and light green triangles representing FhuA, BtuB, LptD and LamB-dependence. Purple triangles show phages isolated in our lab that are not FhuA-dependent but the receptor they target remains unknown. T4 serves as an outgroup so no triangle was given but it has previously been shown to be dependent upon OmpC and LPS (54). To our knowledge, all other phages without a triangle remain unknown.

Table S1. Plasmids and strains used in this study.

Plasmid	Genotype and/or phenotype	Source
pKG116	P15A ori; Cm^r	(104)
pJL002	pKG116 *fhuA* wild type	This study
pJL004	pKG116 *fhuA* ΔL3 (Δ247-273::NSEGS)	This study
pJL005	pKG116 *fhuA* ΔL4 (Δ318-339::NSEGS)	This study
pJL006	pKG116 *fhuA* ΔL5 (Δ394-419::NSEG)	This study
pJL007	pKG116 *fhuA* ΔL8 (Δ552-558::NSEG)	This study
pJL008	pKG116 *fhuA* ΔL10 (Δ640-654::NSEGS)	This study
pJL009	pKG116 *fhuA* ΔL11 (Δ689-701::NSEG+A702S)	This study
pJL010	pKG116 *fhuA* ΔL3L5 (Δ247-273::NSEGS), (Δ394-419::NSEG)	This study
pJL011	pKG116 *fhuA* ΔL4L5 (Δ318-339::NSEGS), (Δ394-419::NSEG)	This study
pJL012	pKG116 ΔL5L8 (Δ394-419::NSEG), (Δ552-558::NSEG)	This study
pKD46	Lambda red recombinase	(63)
pCP20	Flp recombinase	(105)
**Strains**		
MG1655	F^-,λ^-, *rph-1*	(106)
MG1655	pKD46	This study
MG1655	Δ*fhuA*::*kan*	This study
MG1655	Δ*fhuA*::*kan* pCP20	This study
MG1655	Δ*fhuA*::FRT	This study

MG1655	Δ*fhuA*::*kan* pKG116	This study
MG1655	Δ*fhuA*::*kan* pJL002	This study
MG1655	Δ*fhuA*::FRT Δ*fepA*::*kan* pKG116	This study
MG1655	Δ*fhuA*::FRT Δ*fepA*::*kan* pJL002	This study
MG1655	Δ*fhuA*::FRT Δ*fepA*::*kan* pJL004	This study
MG1655	Δ*fhuA*::FRT Δ*fepA*::*kan* pJL005	This study
MG1655	Δ*fhuA*::FRT Δ*fepA*::*kan* pJL006	This study
MG1655	Δ*fhuA*::FRT Δ*fepA*::*kan* pJL007	This study
MG1655	Δ*fhuA*::FRT Δ*fepA*::*kan* pJL008	This study
MG1655	Δ*fhuA*::FRT Δ*fepA*::*kan* pJL009	This study
MG1655	Δ*fhuA*::FRT Δ*fepA*::*kan* pJL010	This study
MG1655	Δ*fhuA*::FRT Δ*fepA*::*kan* pJL011	This study
MG1655	Δ*fhuA*::FRT Δ*fepA*::*kan* pJL012	This study
MG1655	Δ*fhuA* pKG116	This study
MG1655	Δ*fhuA*::FRT *ΔwaaC*::*kan* pJL002	This study
MG1655	Δ*fhuA*::FRT *ΔwaaC*::*kan* pJL004	This study
MG1655	Δ*fhuA*::FRT *ΔwaaC*::*kan* pJL005	This study
MG1655	Δ*fhuA*::FRT *ΔwaaC*::*kan* pJL006	This study
MG1655	Δ*fhuA*::FRT *ΔwaaC*::*kan* pJL007	This study
MG1655	Δ*fhuA*::FRT *ΔwaaC*::*kan* pJL008	This study
MG1655	Δ*fhuA*::FRT *ΔwaaC*::*kan* pJL009	This study
MG1655	Δ*fhuA*::FRT *ΔwaaC*::*kan* pJL010	This study

MG1655	Δ*fhuA*::FRT Δ*waaC*::*kan* pJL011	This study
MG1655	Δ*fhuA*::FRT Δ*waaC*::*kan* pJL012	This study

Table S2. List of primers used in this study.

Primer	Sequence (5' to 3')	Source
fhuA KO F	ATCATTCTCGTTTACGTTATCATTCACTTTACATCAGAGATATACCAA TGATTCCGGGGATCCGTCGACC	(107)
fhuA KO R	GCACGGAAATCCGTGCCCCAAAAGAGAAATTAGAAACGGAAGGTTG CGGTTGTAGGCTGGAGCTGCTTCG	(107)
waaC KO F	AGTTTAAAGGATGTTAGCATGTTTTACCTTTATAATGATGATAACTTTT CATTCCGGCGATCCGTCGACC	This study
waaC KO R	TACTGGAAGAACTCAACGCGCTATTGTTACACAAGAGGAAGCCTGAC GGATGTGTAGGCTGGAGCTGCTTCG	This study
k1	CAGTCATAGCCGAATAGCCT	(63)
k2	CGGTGCCCTGAATGAACTGC	(63)
kt	CGGCCACAGTCGATGAATCC	(63)
fhuA cloning F	GCATGTTCCATATGGCGCGTTCCAAAACTGCTC	This study
fhuA cloning R	GCATGTTCGGTACCTTAGAAACGGAAGGTTGCGGTTGC	This study
pKG116 R	CGGGGAAAACTTGCTTTTTCC	This study
FhuA loop 3F	AGGTAGCACCTATTCTCGTAATGAGAAGATG	This study
FhuA loop 3R	TCGCTGTTCCAGCCGTAATAACCGGT	This study
FhuA loop 4F	AGGTAGCTATCTGGCACGTAAATAC	This study

FhuA loop 4R	TCGCTGTTGACGCCGTAACCATAAAC	This study
FhuA loop 5F	GAAGGTTCCGGCCCTTACCGCATT	This study
FhuA loop 5R	GCTGTTGTAACCAAACCAGGCGTTGATG	This study
FhuA loop 8F	GAAGGTTCGGTTGAAGGTGGCGAG	This study
FhuA loop 8R	GCTGTTCGCCATCAGGTTGTTGGTTTTAG	This study
FhuA loop 10F	AGGTAGCTATACGGTCGTGGATGCG	This study
FhuA loop 10R	TCGCTGTTAGTATAACGACCACCGGTG	This study
FhuA loop 11F	AGGTAGCGAACGTCAGGTCGTTGCA	This study
FhuA loop 11R	TCGCTGTTGTATTCACGATCGAACAGG	This study

Table S3 Amino acids included in RBP terminal ends.

Phage	RBP length (amino acids)	N-terminal region	C-terminal region
T4	1026	1-700	701-1026
T5	640	1-428	429-640
IrisVonRoten	585	1-400	401-585
IME178	1226	1-845	846-1226
GEC_vB_N5	949	1-677	678-949
OSYSP	949	1-677	678-949
phiLLS	949	1-677	678-949
vB_EcoD_Pubbukkers	1139	1-776	777-1139
bob	1139	1-776	777-1139
UAE_MI-01	1139	1-776	777-1139

CEC_Kaz_2018	1139	1-776	777-1139
vB_EcoS_PTX06	1140	1-776	777-1140
SECphi4	1139	1-776	777-1139
vB_EcoD_Teewinot	1139	1-776	777-1139
JLBYU37	1139	1-776	777-1139
Envy	1139	1-776	777-1139
slur05	1050	1-776	777-1050
vB_SboD_StarDew	1148	1-776	777-1148
EK99P-1	1148	1-776	777-1148
HK578	1150	1-776	777-1150
SO1	1148	1-776	777-1148
JLBYU60	1139	1-776	777-1139
PGN590	748	1-641	642-748
slyngel	1141	1-767	768-1141
herni	1141	1-767	768-1141
vojen	1141	1-767	768-1141
pSf-1	1150	1-767	768-1150
JLBYU41	1192	1-767	768-1192
vB_EcoD_Fulano1	1192	1-767	768-1192
JLBYU07	1192	1-767	768-1192
JLBYU26	1192	1-767	768-1192
vB_EcoD_Poky	1192	1-767	768-1192

JLBYU10	1192	1-767	768-1192
JLBYU19	1192	1-767	768-1192
JLBYU01	1192	1-767	768-1192
JLBYU16	1192	1-767	768-1192
vB_EcoD_Phunderstruck	1192	1-767	768-1192
lambda	1132	1-730	731-1132
phi80	1192	1-803	804-1192
ADB-2	851	1-479	480-851
T1	1172	1-767	768-1172
Eco_BIFF	1139	1-767	768-1139
SH6	1149	1-767	768-1149
pSf-2	1149	1-767	768-1149
Sfin-3	1139	1-767	768-1139
